# Signaling pathways in cancer metabolism: mechanisms and therapeutic targets

**DOI:** 10.1038/s41392-023-01442-3

**Published:** 2023-05-10

**Authors:** Mengshu You, Zhuolin Xie, Nan Zhang, Yixuan Zhang, Desheng Xiao, Shuang Liu, Wei Zhuang, Lili Li, Yongguang Tao

**Affiliations:** 1 Hunan Key Laboratory of Cancer Metabolism, Hunan Cancer Hospital and The Affiliated Cancer Hospital of Xiangya School of Medicine, Central South University, 410078 Changsha, Hunan China; 2 NHC Key Laboratory of Carcinogenesis (Central South University), Cancer Research Institute and School of Basic Medicine, Central South University, 410078 Changsha, Hunan China; 3 Department of Pathology, Key Laboratory of Carcinogenesis and Cancer Invasion, Ministry of Education, Xiangya Hospital, Central South University, 410078 Changsha, Hunan China; 4 Department of Pathology, Xiangya Hospital, Central South University, 410008 Changsha, Hunan China; 5 Department of Oncology, Institute of Medical Sciences, Xiangya Hospital, Central South University, 410008 Changsha, Hunan China; 6 Department of Thoracic Surgery, Xiangya Hospital, Central South University, 410008 Changsha, Hunan People’s Republic of China; 7 Cancer Epigenetics Laboratory, Department of Clinical Oncology, State Key Laboratory of Translational Oncology, Sir YK Pao Centre for Cancer and Li Ka Shing Institute of Health Sciences, The Chinese University of Hong Kong, Ma Liu Shui, Hong Kong; 8 Department of Thoracic Surgery, Hunan Key Laboratory of Early Diagnosis and Precision Therapy in Lung Cancer, Second Xiangya Hospital, Central South University, 410011 Changsha, China

**Keywords:** Cancer metabolism, Cancer microenvironment

## Abstract

A wide spectrum of metabolites (mainly, the three major nutrients and their derivatives) can be sensed by specific sensors, then trigger a series of signal transduction pathways and affect the expression levels of genes in epigenetics, which is called metabolite sensing. Life body regulates metabolism, immunity, and inflammation by metabolite sensing, coordinating the pathophysiology of the host to achieve balance with the external environment. Metabolic reprogramming in cancers cause different phenotypic characteristics of cancer cell from normal cell, including cell proliferation, migration, invasion, angiogenesis, etc. Metabolic disorders in cancer cells further create a microenvironment including many kinds of oncometabolites that are conducive to the growth of cancer, thus forming a vicious circle. At the same time, exogenous metabolites can also affect the biological behavior of tumors. Here, we discuss the metabolite sensing mechanisms of the three major nutrients and their derivatives, as well as their abnormalities in the development of various cancers, and discuss the potential therapeutic targets based on metabolite-sensing signaling pathways to prevent the progression of cancer.

## Introduction

Since Warburg discovered aerobic glycolysis as a metabolic marker of cancer cells, extensive studies have enhanced our understanding of the metabolic changes in cancer cells.^1–5^ Metabolic reprogramming is a common feature of many tumors, enabling cancer cells to meet their specific growth requirements through alterations in glucose, lipid, and amino acid metabolism.^6,7^ In addition to the well-known phenomenon of aerobic glycolysis, cancer-specific metabolic changes have been discovered, including elevated lactate production, increased glutamine metabolism, fatty acid synthesis, and decreased fatty acid oxidation.^1,2^ The cellular metabolism of tumors is a complex and diverse spectrum, with each nutrient’s source and metabolic pathways being better understood.^8^

Metabolic reprogramming satisfies the growing demands of cancer cells and governs their behaviors by modulating the types and concentrations of metabolites in the tumor microenvironment.^9^ In addition to endogenous metabolites, exogenous nutrients also impact the metabolic microenvironment of tumors. Cells possess the ability to sense changes in metabolism and initiate a cascade of reactions known as metabolite sensing, which involves the regulation of cell signaling transductions and epigenetic modifications.^7^ Metabolite sensing enables changes in metabolites to be translated into biochemical signals that regulate a series of signal transduction pathways and gene expression in cells. Metabolite sensing serves as a crucial link between the external environment and cells, allowing cells to promptly detect changes in the external environment, reorganize the metabolic network, and adjust cell signaling and other cellular processes.^10^ However, some of these changes may promote cancer progression.^11^

Metabolite sensing can be achieved in three ways: metabolite sensor-mediated signal transduction, a metabolic sensing module, and conjugate sensing. Metabolite sensors are biological macromolecules, including proteins, RNAs, and DNAs, that directly bind to metabolites, triggering changes in downstream proteins. These sensors possess three key characteristics: 1. Specificity: Specificity is achieved using specific domains that enable sensors to recognize and bind to metabolites with high specificity, ensuring accurate metabolite sensing.^12^ 2. Dynamicity: Dynamicity refers to the reversible nature of the sensor-metabolite interaction, allowing sensors to sense fluctuations in metabolite concentrations and enabling competitive metabolite binding assays to verify sensor identity.^13,14^ 3. Functionality: Functionality is achieved by changes in the activity or function of metabolic receptors sensors, as the different states of metabolites alter protein conformation or interaction. It is worth noting that the role of receptor sensors is to translate the chemical signal of metabolites into a biological signal that interacts with the biological networks, thereby acting as translators of the cellular environment.

Epigenetic modifications are important in many diseases, especially in cancer development.^15^ Epigenetics regulates gene expression without altering the DNA sequence and occur at multiple levels, including chromatin remodeling, DNA modification, histone protein modification, non-coding RNA regulation, and nucleosome positioning.^16^ Epigenetic alterations related to oncogenes and tumor suppressor genes often cause cell transformation, tumor progression, and metastasis.^17^ For instance, lymphoid-specific helicase (LSH), a chromatin remodeling factor that regulates DNA methylation patterns, has been found to play a crucial role in epigenetics.^11,18^ LSH is highly expressed in almost all B-cell lymphoma samples^19^ and has been implicated in the pathogenesis of other tumors, such as glioma, lung cancer, and nasopharyngeal carcinoma.^20^ Metabolites also contribute to chromatin dynamics through chemical posttranslational modifications (PTMs) that alter chromatin structures and functions, in addition to acting as signaling molecules to respond to the environment via metabolite sensing mechanisms.^21^ Histones can undergo a wide range of PTMs, such as acetylation, methylation, phosphorylation, and other acylation modifications, as DNA and RNA are chemically modified by methylation. In general, cancer cell proliferation and distant metastasis are related to epigenetic changes, including increased histone acetylation and decreased histone methylation,^22^ while DNA methylation is linked to gene silence,^23^ histone acetylation is related to gene transcription.^24^ Currently, there are two main viewpoints regarding the role of metabolites in epigenetic regulation: one suggests that metabolites provide substrates for epigenetic enzymes, while the other proposes that metabolites act as allosteric regulators of these enzymes. For example, in HeLa cells, acetic acid absorbed from the culture medium can be covalently bound to histones, leading to histone acetylation and the regulation of protein function.^25^ This finding supports that metabolites can covalently modify proteins and regulate their functions. In addition, increased succinate inhibits histone demethylase activity, triggering.^26^ Recently, epigenetic drugs have received extensive attention, such as histone demethylases (HDM) inhibitors, bromodomain and extra-terminal motif (BET) inhibitors, and histone deacetylase (HDAC) inhibitors. However, these drugs have been ineffective on solid tumors due to stability and dose limitations. As a result, combination therapy has emerged as a promising avenue in epigenetic therapy, with ongoing clinical trials exploring the use of programmed death-1 (PD-1) or programmed death ligand-1 (PD-L1) mAbs in combination with DNA methyltransferase (DNMT), HDAC and enhancer of enhancer of zest homolog 2 (EZH2) inhibitors are in clinical trials.

In humans, cell metabolism is integrated into a complex biological network that involves a wide range of metabolites and ubiquitous metabolite-sensing mechanisms. Endogenous or exogenous metabolites can regulate signal transduction and epigenetics of these mechanisms. However, aberrant metabolic reprogramming in cancers can lead to abnormal inhibition and activation of metabolite sensing, which is a significant contributor to cancer progression. Thus, the abnormality of metabolite sensing suggests the existence of numerous potential targets that could be targeted to suppress tumorigenesis and cancer development.^27^ Meanwhile, epigenetic regulation in cancer development is a post-genomic era revolution in cancer genetics, which may provide new targets for cancer therapy.^28^ Interestingly, unlike genetic mutations, epigenetic mutations are reversible, which means that the epigenome can be reprogrammed and has high potential as a therapeutic strategy for cancer.^29^

In this review, we focus on the effect of the tumor metabolic microenvironment on cancer cells, especially on how metabolites influence signal transduction and epigenetics in cancer cells. A better understanding of the tumor metabolic microenvironment’s effect on cancer cells’ life activities will be performed and some potential therapeutic targets based on the tumor metabolic microenvironment will be discussed.

## Signal transduction and epigenetic regulation of glucose metabolism

### Signal transduction mediated by GPCRs

G protein-coupled receptors (GPCRs) constitute the most prominent family of receptors in mammals and are involved in regulating almost all cellular and physiological functions in the body. These receptors have gained attention as targeted drug development candidates due to their high specificity and affinity for ligand binding, accounting for approximately 20% of currently developed drug targets.^30^ Once activated by the ligand, GPCR can be coupled to four different heterotrimeric G protein families (Gs, Gi/Go, Gq/G11, and G12/G13) and then act on different effectors, such as downstream enzymes and ion channels.^31–33^ Therefore, the modular structure of the signaling system mediated by GPCR and G protein is vital for its role. In GPCRs, there are several receptors worth paying attention to, and the following are related to glucose metabolism:

#### GPR31 plays an essential role in the immune system and tumor progression

GPR31 is a G protein-coupled receptor that recognizes citric acid cycle intermediates. In addition to its critical role in inflammation, emerging evidence suggests that GPR31 contributes to tumor progression.^34^ Recent studies have identified lactic acid and pyruvate as potent inducers of GPR31-mediated dendritic processes of intestinal CX3CR1 + phagocytes, potentially enhancing the immune response. These findings suggest that GPR31 may play a complex role in cancer development and progression and may represent a promising target for anti-tumor therapy. Further research is needed to fully elucidate the mechanisms underlying GPR31’s functions and to explore its therapeutic potential.^35^

#### The connection between SUCNR1 and the cancer metastasis

Succinate receptor 1(SUCNR1), also known as GRP91, is widely expressed in different organs. SUCNR1 is activated by succinate and as an intermediate molecule of the citric acid cycle. Studies have shown that SUNCR1 plays an essential role in tumor metastasis, especially in individuals with succinate dehydrogenase (SDH) germline mutation. Extracellular succinate activated the PI3K-Akt pathway by combining SUCNR1 in tumor cells and upregulating HIF-1α, promoting cancer cell invasion and driving epithelial–mesenchymal.^36–38^ In gastric cancer, it was found that succinate could activate it via the SUCNR1-ERK1/2-STAT3-VEGF pathway leading to the angiogenesis.^39^ Besides driving cancer cell migration, extracellular succinate significantly impacts macrophages within the tumor microenvironment. Succinate induces polarization of tumor-associated macrophages (TAMs) by activating the SUCNR-1 receptor on macrophage membranes and its downstream PI-3K/Akt-HIF-1α signaling pathway. This succinate-induced macrophage polarization leads to increased cancer cell migration by promoting the secretion of promigratory cytokines, such as interleukin (IL) -6. Moreover, extracellular succinate targets M2 macrophages and activates M2 macrophage gene transcription through SUCNR1.^40^ SUCNR1 expression is upregulated in human SDH-mutated tumors and in various prevalent cancers. It may be associated with heightened risks of tumor metastasis and recurrence, making it a potential predictive biomarker for SDH-mutated tumors.

### The function of glucose and its metabolites in epigenetics regulation

#### Ubiquitination and acetylation, and tyrosine phosphorylation of histone H3 induced by glucose

Histone protein is an important part of epigenetic regulation. It’s also the central component of the nucleosome which is the element responsible for the stable maintenance of repressive chromatin. The nucleosome is composed of histones H2A, H2B, H3, and H4 in an octameric core with a linker histone, H1.^15^ Histone H3 ubiquitination markers are essential in transcriptional regulation. H3 ubiquitination is physiologically induced by glucose, and H3 acetylation has also been extensively studied and has been shown to occur at specific lysine residues, including K9, K14, K27, and K56 by selectively recruiting histone acetyltransferases (HAT) general control non-depressible 5 (GCN5). Whole genome chromatin immunoprecipitation and sequencing (ChIP-seq) data set analysis showed that glucose-induced H3 acetylation in a gene-specific manner (approximately 2000 genes) at the transcription start site (TSS). The comprehensive analysis combined with ChIP-seq and gene expression microarray data sets further indicates that these acetylation events at TSS are significantly related to the expression of their target genes, many of which are involved in cancer-related pathways in the neuronally expressed developmentally down-regulated 4 (NEDD4)-dependent manner. Interestingly, H3 ubiquitination and NEDD4, GCN5, and histone H3 are essential regulators of tumor formation. Glucose-induced H3 ubiquitination target genes, such as IL-1α, IL-1β, and glutamate-cysteine ligase modifier (GCLM) subunit, are also important factors for tumor sphere formation.^41^

Glucose can induce the tyrosine phosphorylation of NEDD4, thereby activating the activity of NEDD4 E3 ligase. However, it needs to be made clear which upstream signaling pathway is involved in NEDD4 phosphorylation. While the upstream signaling pathway involved in NEDD4 phosphorylation is not entirely understood, previous studies have shown that growth factors such as fibroblast growth factor or epidermal growth factor can induce NEDD4 tyrosine phosphorylation through Src kinase.^42^ Instead, it has been suggested that the tyrosine kinase. Yes, the closest member of the Src family kinase (SFK) to Src may be involved in glucose-induced NEDD4 phosphorylation. Previous studies have also shown that glucose activation of SFK is essential for glucose-induced NEDD4 activation. This suggests that SFKs may be involved in the upstream signaling pathway that leads to NEDD4 phosphorylation in response to glucose.^43^

Hyperglycemia or hyperglycemic state can affect cancer development and progression. Studies have shown that hyperglycemic conditions can promote a more aggressive phenotype in various cancers, including breast and liver cancer. In diabetic patients, hyperglycemia is an important cause of increased cancer mortality. High glucose conditions increased the phosphorylation of histone H3 at serine 10, which is associated with increased cell proliferation.^44^

#### The preferential expression of PKM2 protein promotes the increase of glycolysis

The preferential expression of Pyruvate kinase isozyme M2 (PKM2) is related to increased aerobic glycolysis and the growth advantage of cancer cells.^45^ After the depletion of DNA methyltransferase three beta (DNMT3β) or Brother of the Regulator of Imprinted Sites (BORIS) and the mutation of Bardet–Biedl syndrome (BBS) at exon 10 of PKM, the splicing transition from PKM2 to PKM1 isomer is related to the reversal of the Warburg effect, which further inhibits the growth of breast cancer cells. Although the observed reversal of the Warburg effect may not be entirely due to the splicing transition caused by DNMT3β and BORIS, it can partially explain the cause of poor prognosis or poor growth of breast cancer associated with DNMT3β^46^ and BORIS.^47^

#### The effect of glucose on OGT and O-GlcNAcylation

High glucose levels in cells have been found to increase the concentration of UDP-*N*-acetylglucosamine (UDP-GlcNAc), which in turn increases the overall O-β-N-acetylglucosamine (O-GlcNAc) levels.^48^ O-GlcNAcylation refers to an O-GlcNAc moiety that attaches to a serine or threonine residue on nuclear or cytoplasmic proteins.^49^ The addition and removal of GlcNAc to proteins are catalyzed by O-GlcNAc transferase (OGT) and O-GlcNAcase (OGA), respectively.^50^ O-GlcNAcylation regulates a variety of cellular processes, such as nutritional sensing, cell cycle progression, transcription, translation, epigenetic regulation, and protein-protein interactions.^50–53^ Glycosyl groups of O-GlcNAc are considered to mediate glucose homeostasis and stress response, serving as a signaling cascade.^54^

**Fig. 1:**
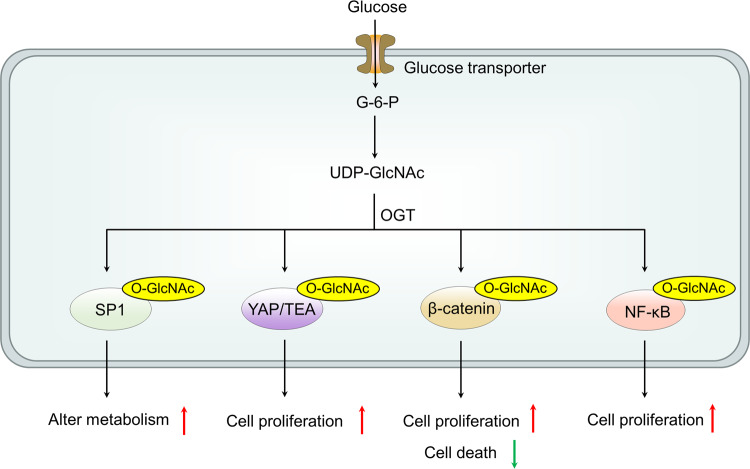
The effect of glucose on OGT and O-GlcNAcylation. GlcNAc is an O-β-N-acetylglucosamine moiety attached to a nuclear protein, cytoplasmic protein serine, or threonine residue. The addition of GlcNAc to proteins is catalyzed by OGT, while its removal is catalyzed by OGA. Currently, several pathways have been proposed that implicate O-GlcNAcylated proteins in the link between tumorigenesis and hyperglycemia. O-GlcNAcylation stabilizes SP1, β-catenin and YAP proteins promoting expression of target genes and transcriptional activity of NF-kB pathway. O-GlcNAc O-β-*N*-acetylglucosamine, OGT O-GlcNAc transferase, OGA O-GlcNAcase, SP1 Specificity protein 1, YAP Yes-associated protein

Elevated O-GlcNAcylation levels have been observed in different types of cancer; It is noteworthy that the inhibition of OGT has been demonstrated to impede tumor growth in various models.^55–57^ Among many eukaryotic initiation factors in the translation mechanism, eukaryotic initiation factor 4E (eIF4E) is a key factor regulating the initiation of translation, which is also modified by O-GlcNAc in liver cancer.^53^ O-GlcNAcylation has been shown to be involved in regulating protein stability. Specifically, at T168/T177, O-GlcNAcylation protects eIF4E from degradation, resulting in increased eIF4E protein levels. Given the fact that the high expression of eIF4E indicates a poor prognosis of HCC, the OGT-eIF4E axis plays a critical role in the progression and prognosis of HCC. Moreover, OGT activates the stem cell potential of HCC cells by upregulating eIF4E. These results indicate that glucose promotes the proliferation of hepatoma cells and stem cell-like potential through OGT. Currently, several pathways have been proposed that implicate O-GlcNAcylated proteins in the link between tumorigenesis and hyperglycemia. Specificity protein 1 (Sp1) is an important transcription factor in cells. In cancer, higher levels of Sp1 are associated with tumor cell survival, proliferation and invasion, and angiogenesis within the tumor. Sp1 is O-GlcNAcylated, and at least one O-GlcNAc site is associated with its stability.^58^ Therefore, Hyperglycemia and high glucose increase O-GlcNAcylation of Sp1, thereby promoting tumor cell metabolism and survival.^59^ Elevated glucose levels enhance the transcriptional activity of at least two members of the NF-kB family, p65 and c-Rel, via O-GlcNAcylation modification. The NF-kB pathway is known to be associated with the Hexosamine Biosynthetic Pathway, which has been shown to promote the growth of tumor cells.^60^ Similar to its role in regulating NF-kB, high glucose conditions increase the activity of the β-catenin pathway, amplifying the response of different human cancer cells to Wnt through O-GlcNAcylation.^61^ O-GlcNAc has been found to stimulate Yes-associated protein (YAP) function, which plays a crucial role in tumorigenesis. When GlcNAc and PUGNAc induce Hyper-O-GlcNAcylation or when OGT is overexpressed, the transcriptional activity of YAP/TEAD increases along with the expression of their target gene CTGF, which is related to cell proliferation^62^ (Fig. 1).

#### Glucose starvation suppresses histone 2A K119 monoubiquitination (H2Aub)

Glucose starvation inhibits H2Aub, a histone modification related to gene suppression.^63–65^ The inhibition of H2Aub levels by glucose deficiency is independent of energy stress-mediated AMP-activated protein kinase(AMPK) activation and may result from nicotinamide adenine dinucleotide phosphate (NADPH) depletion and subsequent inhibition of BMI1, a component of polycomb repressive complex 1 (PRC1) that catalyzes H2Aub on chromatin sections.^66–71^ Comprehensive transcriptomic and epigenomic analysis link glucose starvation-mediated H2Aub suppression with the activation of genes related to the endoplasmic reticulum (ER) stress response.^72^ The epigenetic mechanism plays a role in glucose starvation-induced cell death, and the pharmacological inhibition of glucose transporter 1 (GLUT1) and PRC1 synergistically promotes ER stress and inhibits tumor growth in vivo. Together, these results reveal a hitherto unrecognized epigenetic mechanism that couples the availability of glucose with the ER stress response.^73^

#### The role of ncRNA in the glucose metabolism

Non-coding RNA (ncRNA) is a class of functional RNA molecules that regulate the expression of target genes without being translated into proteins. Based on their size, ncRNAs can be classified into small ncRNAs (including microRNA, small interfering RNA, PIWI-interacting RNA, small nucleolar RNA, and small nuclear RNA) and long ncRNAs(lncRNA). The transcription and processing method of lncRNA is similar to that of microRNA. Functionally, lncRNA exerts various mechanisms of action in cells, including direct transcriptional regulation, histone modification, and regulation of the transcription of regulatory factors acting as bait-binding sites. Gene expression profiles indicate that lncRNA is expressed in a tissue-specific or cell-specific manner and has different expressions under different pathophysiological conditions. ncRNA is associated with a variety of cancers. mircoRNA and lncRNA, known as oncogenes and tumor suppressors, act on receptor tyrosine kinase (RTK) and hypoxia-mediated pathways. Anticancer microRNA replacement therapy has a high potential in cancer treatment. For example, the drugs for bone metastasis and colon cancer, which are microRNA mimics, resulted in a significant decrease in tumor size.^74^

ncRNAs modulate glucose transport in cancer cells by elevating GLUT levels. Among the 14 GLUT isotypes,^75,76^ GLUT1 is known to be overexpressed in cancer. ncRNA is involved in the regulation of GLUT1 expression. The lncRNA neighbor BRCA1 gene 2 (NBR2) promotes cell survival by upregulating GLUT1 expression.^77^

In addition to regulating GLUTs, ncRNAs also modulate key glycolytic enzymes involved in the three critical steps of glycolysis. The first is the phosphorylation of glucose by hexokinases (HKs) to produce glucose 6-phosphate (G6P). In addition to the ubiquitously expressed HK1, cancer cells overexpress HK2, which is critical to the Warburg effect because phosphorylated glucose is trapped in the cytoplasm. Other regulators of HK2 are microRNA-143 and -145, which cluster on chromosome 5q32 and are down-regulated in many cancer types.^78,79^ The second key step of glycolysis is the conversion of fructose-6-phosphate (F6P) to fructose-1,6-bisphosphate (F1,6P) under the catalysis of phosphofructokinase 1 (PFK1). TAT-activated regulatory DNA binding protein (TARDBP), which is highly expressed in hepatocellular carcinoma (HCC), regulates glycolysis by inducing the expression of the platelet isoform PFK1 (PFKP). TARDBP inhibits the expression of microRNA-520a/b/e by directly binding to its promoter region, preventing it from binding to the 3’-UTR of PFKP and thereby inhibiting protein expression. The last key step of glycolysis is catalyzed by pyruvate kinase (PK). Cancer cells use various functional gain strategies to increase glycolysis. Although PK function can be attenuated by expressing the low-affinity dimer form of PKM2, the tetrameric form of PKM2 and PKM1 play a role in the physiological phosphoenolpyruvate level of normal cells. Some have found that microRNA expression PKM2 adjustment disorders in cancer. microRNA-133a/b is downregulated in several types of cancer.^80,81^ microRNA-326 displayed direct targets and inhibited the expression of PKM2. In addition, microRNA-326 levels and high levels of glioma cells PKM2 are negatively correlated, indicating endogenous PKM2 adjustment mechanism.^82^

Many ncRNAs have been found to regulate PI3K/Akt/mTOR signaling. We are here to focus on ncRNAs known to be associated with the Warburg effect. microRNA-451 can down-regulate the expression of GLUT1 in glioma cell lines by inhibiting the PI3K/Akt pathway and glycolysis.^83^ LncRNA Maternally Expressed Gene (MEG) 3 is expressed in various normal tissues and is silenced in several primary human tumors and cell lines. Ectopic expression of MEG3 leads to the accumulation of p53 and altered expression of p53 target genes in tumor cells, leading to growth inhibition. These findings indicate that MEG3 can negatively regulate glycolysis through p53 and act as a tumor suppressor.^84,85^

#### The target therapy concentrated on glucose metabolite

So far, there are several target points that may be correlated with the therapy. The primary approach is targeting GLUTs. It included several preclinical glycolytic inhibitors such as phloretin, fasentin, STF-31, WZB117, ritonavir, and silybin.^86^ The silybin has entered Phase I clinical trial.^87^ Another approach is to target HK enzymes, with 2-deoxy-D-glucose (2-DG),^88,89^ and lonidamine (LN)^90^ currently under Phase II clinical trial, and resveratrol, genistein-27, benserazide, astragalin, and chrysin under preclinical research.^91–95^ However, targeting HK2 in anticancer therapy is challenging due to the toxicity of normal cells and the high doses required by current inhibitors. Additionally, targeting specific HK subtypes is difficult due to the highly conserved domains of HK1 and HK2. Targeting PI3K/Akt/mTOR pathway is another option, with many inhibitor agents under testing in preclinical and early clinical studies as targeted anticancer therapies. These agents include Afuresertib, Uprosertib, and Ipatasertib.^96^ However, the single therapy is only efficacy in malignancy with PIK3 mutation or PTEN deficiency.^97^ In addition, mTOR inhibitors have shown clinical benefits in several tumors, such as neuroendocrine, endometrial, and breast cancers.^98,99^ Rapalogs, a mTORC1 inhibitor, shows limited activity in clinical trials as single anticancer agents, probably due to the various cross-talks of the complicated mTOR pathway with other signaling pathways.^100^ The newer generations of dual mTOR kinase inhibitors (PP242, NVP-BEZ235) are less liable to induce tumor resistance than the rapalogs. These agents are tested in preclinical studies and recently entered some clinical trials.^101,102^ However, the great metabolic heterogeneity and cellular plasticity observed in solid tumors make metabolic inhibitors unlikely to become effective as monotherapy for cancer. Therefore, combination therapy is the future area of focus.

## Metabolite sensing and epigenetic regulation of fatty acid metabolism

With the concept of tumor metabolism micro-environment, fatty acid metabolism in cancers have been increasingly focused on. The oxidation of fatty acids provides energy in the form of ATP and NADH, while its synthesis provides the foundation for cell structure.^103^ To meet the specific growth needs of tumor cells, there are increased fatty acid uptake and synthesis, as well as decreased fatty acid oxidation in cancer cells.^1,2^ Regulating abnormal fatty acid metabolism inhibits tumorigenesis and improves cancer-free survival.^104,105^ Currently, there are many anti-cancer drugs targeting fatty acid metabolism, such as FASN inhibitors, ASSC2 inhibitors and so on.^106^ Meanwhile, due to the changes in fatty acid metabolism in cancers, certain specific fatty acids may serve as potential biomarkers for cancer diagnosis.^107,108^

Cells convert abnormal fatty acids concentration and categories into a series of biochemical information through metabolite sensor-mediated signal transduction and metabolite conjugate sensing mechanism.^7,104^ Also, fatty acids and their intermediate metabolites play important roles in the origination and development of cancer by regulating epigenetic mechanisms. With the deepening of research, some new small molecule tools are constantly improving, which provides the possibility for in vivo lipidomics.^109^

### Signal transduction of fatty acid and its derivatives

#### GPCR is a recognizer of fatty acid and its derivatives

Sensors of fatty acids and their intermediate metabolites are mainly various types of GPCRs, such as GPR41, GPR43, GPR109A, GPR78, GPR84, GPR120, etc. Among them, GPR41, GRR43, and GPR109A recognize SCFAs. GPR43 prefer short-chain fatty acids, acetate, and propionic acid.^110^ GPR41 mainly binds to amyl acetate, butyrate, and propionate. GPR109A and GPR109B show 96% identity at the protein level. In short-chain fatty acids (SCFAs), only butyrate activates GPR109A,^111,112^ while any short-chain fatty acids can activate GPR109B.^111^ GPR84 recognizes medium-chain fatty acids(C9-C14).^113^ GPR120 recognizes ω-3 fatty acids and long-chain fatty acids.^114,115^ GPR131 can identify primary and secondary (bacterial origin) bile acid metabolites.^116^

**Fig. 2:**
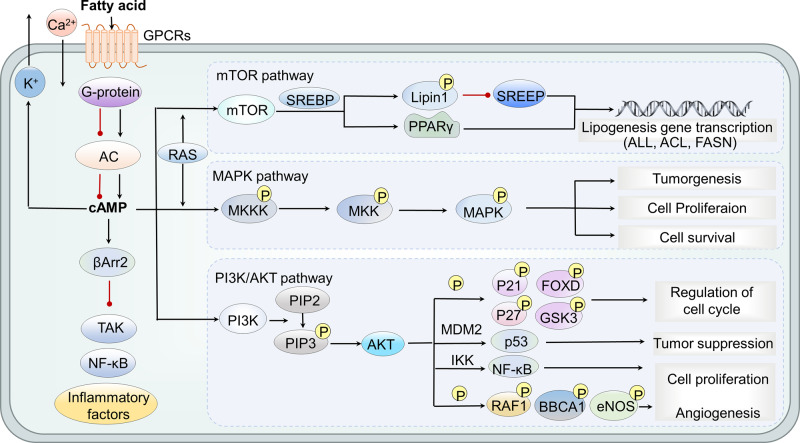
GPCRs-mediated short-chain fatty acid signal transduction. Dietary fiber in the diet can produce short-chain fatty acids under the action of intestinal microorganisms. Short-chain fatty acids can activate the corresponding GPCRs to cause a series of downstream signaling and trigger a variety of biological functions. GPCRs activation can induce changes in cell shape and movement, induce Ca^2+^ or K^+^ efflux, or trigger downstream PI3K/MTOR/MAPK pathways. PI3K can phosphorylate PIP2 to PIP3 which can activate AKT, promoting cell proliferation and angiogenesis by phosphorylating different factors, regulate cell cycle, inhibiting apoptosis and regulating NF-κß and p53 signaling pathway. mTORC1 phosphorylates Lipin1, inhibits SREBP1/2-dependent transcription, and increases PPARγ activity, thereby increasing the transcription of ACC, ACLY, and FASN. The MAPK pathway maintains the expansion of key molecules in the process of cell proliferation, growth and survival, and promotes EMT of cancer cells. RAS is not only an activator of MAPK pathway, but also an activator of P13k/AKT/mTOR pathway. In addition, GPCR can also send signals through β-arrestin, which can inhibit the activation of NF-κB and the production of pro-inflammatory cytokines. PI3K phosphatidylinositol 3 kinase, MTOR mitogen-activated protein, PPARγ peroxisome proliferator-activated receptor γ, ACC acetyl-CoA carboxylase, ACLY ATP citrate lyase, FASN fatty acid synthase, EMT epithelial mesenchymal transformation, SREBP sterol regulatory element binding protein

After the metabolites are recognized by their specific sensors, they mainly transmit information through two pathways. The first one is through G protein pathway. Specifically, GPCRs can regulate effectors such as enzymes and ion channels mentioned in the parts of glucose.^31–33^ For example, by activating MAPK, PI3K, mTOR, etc., the changes in cell metabolism can promote the progression of cancers.^117–122^ The MAPK, PI3K, and mTOR signal pathways play important roles in maintaining cell proliferation, growth, and survival,^123,124^ and are often abnormally altered in various cancers (Fig. 2). Notably, mTORC1 complex regulates lipid synthesis through multiple mechanisms by regulating the binding protein of sterol regulatory element-binding proteins (SREBP).^125^ SREBP transcription factors are the main regulators controlling the expression of most fatty acid synthesis enzymes. It phosphorylates Lipin1, preventing it from translocating to the nucleus, thereby inhibiting SREBP1/2-dependent transcription.^126^ Also, it can increase the activity and expression of peroxisome proliferator-activated receptor γ (PPARγ) which is a transcription regulator of abiogenic genes.^127,128^ Through these mechanisms, mTORC1 increases the transcription of adipogenic genes, including key enzymes in fatty acid syntheses, such as acetyl-CoA carboxylase (ACC), ATP citrate lyase (ACLY), and fatty acid synthase (FASN), which are related to the progress of cancers, at the same time, a variety of inhibitors targeting key enzymes of fatty acid synthesis pathway have been developed.^106^ Specifically, GPR41, GPR43, and GPR109A are coupled with Gi/o. After binding with the ligand, they reduce the activity of adenylate cyclase and inhibit cyclic adenosine monophosphate (cAMP) through a pertussis toxin (PTX) sensitive mechanism. Besides, GPR43 also has Gq/11-dependent activity which can contribute to the activation of phospholipase C (PLC)-β and the formation of inositol 1,4,5-triphosphate (IP3). Then, IP3 binds and opens the endoplasmic IP3 gated calcium channel, causing the release of calcium into the cytoplasm.^129–133^

The second pathway is mediated by β-arrestin-2. GPR109A and GPR43 are involved in the binding of β-arrestin, some of which are related to the inhibition of nuclear factor-kappa-gene binding (NF-κB), β-arrestin-2 directly interacts with NF-κB inhibitor (IκBα), preventing the phosphorylation and degradation of IκBα.^134^ However, this pathway is mainly related to anti-inflammatory and desensitizing effects,^118,120^ and its role in cancers is still unclear. Noteworthy, SCFAs and their intermediate metabolites bind to different subunits of G protein or β-arrestins, leading to different results in different cells. For example, GPR109A signaling reduces cAMP levels in adipocytes, while leading to the production of prostaglandin D2 (PGD2) in Langerhans cells and macrophages.^135^

#### Abnormal status of fatty acids signal transduction in cancers

##### GPR109A

GPR109A is a G protein-coupled receptor found on the surface of intestinal epithelium cells and immune cells. Studies have shown that the expression of GPR109A mRNA is suppressed in primary colon cancer tissues and colon cancer cell lines. Also, the expression of GPR109A is significantly higher in acute promyelocytic leukemia cells and lung cancer cells.^136^ Niacin and butyric acid as GPR109A agonists contribute to the ectopic expression of GPR109A in colon cancer cells, inducing cell apoptosis.^112^ Colonic epithelial cells use butyrate produced by gut microbiota to synthesize β-hydroxybutyrate. 3-Hydroxy-3-methylglytaryl-CoA synthetase 2 (HMGCS2) controls the synthesis of ketone body β-hydroxybutyrate, but its activity is inhibited in cancers, resulting in the lower level of β-hydroxybutyrate produced by cancer cells. As an endogenous agonist of GPR109A, β-hydroxybutyrate acts through GPR109A which acts as a tumor suppressor. Specifically, when the synthesis of β-hydroxybutyrate is reduced, the tumor suppressive function of GPR109A is weakened, promoting the progression of cancers.^137^ In the colon cancer model related to inflammation, the number of colon polyps of GPR109A−/− mice is significantly more than wild-type mice under the induction of the same carcinogenic factors.^115^ The deficiency of GPR109A also increases the incidence of colon cancer.^115^ Niacin can inhibit colitis and AOM **+** DSS induced carcinogenesis, and this effect has been shown to depend on GPR109A.^115^ Overall, lower butyrate and β-hydroxybutyrate synthesis in cancers inhibits GPR109A signal transduction and promotes carcinogenesis.

Besides, consumption of dietary fiber is associated with a reduced risk of carcinogenesis in breast cancer, prostate and other cancers, as dietary fiber can be degraded by the gut microbiota into short-chain fatty acids (SCFAs). Breast epithelial cells express GPR109A, whereas primary human breast tumor tissues do not express it. Enhanced expression of GPR109A can induce apoptosis and cell cycle arrest in breast carcinoma cell lines. In addition, GPR109A deficiency leads to early development and metastasis of breast cancer.^138^ Moreover, the knockdown of GPR109A leads to increased proliferation of breast cancer cells and GPR109A activation inhibits tumor growth.^115,138,139^ This phenomenon can be explained by the lack of dietary fiber/SCFAs which leads to the development, differentiation or accumulation of inflammatory immune cells in the intestine and local lymph nodes. These inflammatory cells spread or secrete inflammatory factors, inducing inflammation and enhancing carcinogenesis. Therefore, it is essential to investigate whether dietary fiber or SCFAs inhibit carcinogenesis in organs away from the intestine by inhibiting intestinal pathogenic bacteria and/or inflammatory cells.^140^ At the same time, it indicates a possibility: the lack of dietary fiber/SCFAs inhibits the tumor suppressor signal of GPR109A, which is beneficial to the development of cancer. Based on the above, GPR109A agonists can be used for cancer prevention,^137^ such as β-hydroxybutyrate and dietary fiber. Meanwhile, synthetic GPR109A-specific agonists MK1903,^141^ acifran,^142^ 5-aminonicotinic acid (5-ANA),^143^ GSK256073 (8-chloro-3-pentyl-1H-purine-2,6[3H,7H]-dione)^144^ has been explored for its role in vascular and inflammatory diseases, but no preclinical and clinical studies have demonstrated their effectiveness in tumors. To find a strong targeting GPR109A agonist will provide a huge potential in tumor therapy. But there are still many problems that need to be solved before the clinical application of anti-cancer drugs against this target.

##### HCA1(GPR81)/HCA3(GPR109B)

Hydroxycarboxylic acid receptor (HCA) receptor family includes three members, primarily expressed on adipocytes, whose activation can inhibit lipolysis in these cells. The activator of HCA1 is lactic acid, a glycolysis product; the endogenous agonist of HCA2 is the ketone body-3-hydroxybutyrate (3HB); the endogenous agonist of HCA3 is the intermediate of fatty acid β-oxidation (FAO)—3-hydroxyoctanoate (3HO).^145,146^

The expression of HCA1 and HCA3 mRNA in tissues of human breast cancer patients is significantly increased compared with normal tissue samples and primary breast cancer cells, which means enhanced inhibition of lipolysis of adipocytes. In addition, HCA1 mRNA expression is significantly increased in lung cancer cells and acute lymphoblastic leukemia cells. The metabolism of cancer cells is disrupted by the knockdown of HCA3 and HCA1, resulting in reduced viability reduction and/or cell death. Etomuslim and peroxicillin, the fatty acid β-oxidation inhibitors, can prevent HCA3 knockdown-induced cell death in breast cancer cells.^136^ Similarly, the presence of HCA2 and HCA3 mRNA transcripts has also been demonstrated in LoVo colorectal adenocarcinoma cells,^139^ and HCA1 has been confirmed in several cancer cell types including colon cancer, lung cancer and breast cancer.^147^

HCA1 acts as a lactate receptor, but it can also affect fatty acid metabolism. In mice, HCA1 mediates anti-lipolysis in an insulin-dependent manner.^148^ Due to the high rate of glycolysis in cancer cells, high levels of lactic acid are produced and exported. In this way, a sufficiently high concentration of lactic acid activates HCA1, which subsequently leads to the inhibition of lipolysis and FAO.^147^ The knockdown of HCA1 expression will be accompanied by increased mRNAs of HCA2 and HCA3, serving as secondary metabolic monitoring to detect changes in cellular metabolism.^136^ Lactate acts as a signaling molecule by acting as an agonist for GPR81, involving both autocrine and paracrine mechanisms. In the autocrine pathway, lactate produced by cancer cells activates GPR81 on cancer cells; in the paracrine pathway, lactate produced by cancer cells activates GPR81 on immune cells, endothelial cells, and adipocytes present in the tumor stroma. The ultimate result of GPR81 activation is to promote angiogenesis, immune evasion and chemoresistance.^149^ Lactate dehydrogenase A(LDHA) is the main subtype responsible for lactate production which is upregulated in cancer. LDHA inhibitors have been shown to possess anticancer effects in vitro and in vivo.^150–153^ However, LDHA is necessary for normal biological function, so blockading LDHA as a cancer therapy may have many off-target effects and no clinical studies have been carried out. Silencing GPR81 significantly reduced the expression of lactate transporters monocarboxylate transporter 1 (MCT1) and monocarboxylate transporter 4 (MCT4) and their chaperones protein CD147 in pancreatic cancer cell lines.^154^ CD147 has also been proven as a potential drug target to disrupt MCT membrane insertion and function for cancer therapy but also has limitations due to its necessity for normal activities.^155^

Knockdown of HCA3 induces a significant cell death and cell viability reduction in three breast cancer cells: BT-474, HCC1954 and HCC38.^136^ Here is one possible explanation: cancer cells shunt glucose to anabolic processes instead of oxidizing glucose to produce ATP, then energy needs must be met in other ways, such as FAO.^2^ By increasing lipolysis and subsequent FAO, acetyl-CoA which can enter the citric acid cycle is produced.^2^ When the level of acetyl-CoA exceeds the need of the citric acid cycle, ketone bodies (such as 3HB) are generated. Within the presence of glucose, by eliminating this negative feedback mechanism, cancer cells can maintain a high proliferation rate as well as produce ketone bodies, which can be used again as an energy source.^156^ According to Ahmed and others, HCA3 is a sensor of a raised level of 3HO, which is an indicator of a high β-oxidation rate. Activation of HCA3 inhibits the release of free fatty acids, thereby reducing its availability for FAO. In this way, it constitutes another negative feedback mechanism to control lipolytic activity.^146,157^ HCA3 plays a central role in controlling the balance of lipid/fatty acid metabolism in breast cancer cells. Knockdown of HCA3 leads to an uncontrollable increase of FAO in cells, which is the cause of breast cancer cells death. There are few studies on GPR109B antagonists which is worthy of further exploration.

##### GPR43

The expression of GPR43 is lost in human colon cancer cell lines and sharply down-regulated in colorectal adenocarcinoma.^158^ GPR43 deficiency promotes the development of both colon adenoma in ApcMin + DSS (dextran sulfate sodium) mice and the adenomas to adenocarcinoma in azoxymethane/ dextran sulfate sodium (AOM/DSS) mice.^159^ GPR43 deficiency or lacking of fiber in diet can lead to increased recruitment and migration of neutrophils,^160^ which cause inflammation. There are two explanations for the specific mechanism: First, SCFAs may affect the neutrophil L-selectin shedding through GPR43.^161^ However, there are also experimental results that showed SCFA treatment can increase L-selectin expression and L-selectin mRNA levels on the surface of neutrophils,^162^ which may be related to the cancer-promoting effect caused by GPR43 deficiency. It also indicates that acetate salt has potential therapeutic effects for diseases that need to control the neutrophil influx. The second one is that the loss of GPR43 leads to the destruction of the intestinal barrier integrity which promotes the failure of CD8^+^ T cells and the excessive activation of dendritic cells(DCs), leading to the death of mice and promoting the occurrence of colon cancer in mice.^163^ GPR43 deletion promotes the carcinogenesis of colon cancers in mice by reducing the integrity of the intestinal barrier, increasing the bacterial burden of cancers, and changing the phenotype and function of dendritic cells and CD8^+^ T lymphocytes.^163^ Second, GPR43 can differently activate protein kinase B (AKT) or extracellular regulated protein kinases (ERK) signals, and increase the regulation of IL-22 produced by innate lymphoid cell 3 (ILC3). Acetate can enhance the expression of IL-1 receptor (IL-1R) through GPR43, promoting ILC3 to produce IL-22 after stimulation with IL-1β.^164^ Also, GPR43 can promote the expansion of ILC3 which is essential for intestinal homeostasis and host defense.^165^ But its exact role in cancers remains to be further investigated.^166^ In summary, SCFAs can participate in anti-cancer tumor immunity through GPR43, and combine the metabolic microenvironment with the immune microenvironment, which will be a field that worth exploring. Several GPR43 agonists have been reported, such as 4-chloro-α-(1-methylethyl)-N-2-thiazolylbenzeneacetamide (4-CMTB) and AZ1729, but only in preclinical studies in the field of inflammation, its role in cancers is still unknown.^167–170^

##### GPR84

GPR84 is the recognizer of medium-chain fatty acids (C9–C14) including capric acid (C10), undecanoic acid (C11), and lauric acid (C12).^113^ The levels of GPR84 expression are significantly upregulated in human and mouse acute myeloid leukemia (AML) leukemic stem cells (LSCs) compared to normal hematopoietic stem cells (HSCs). GPR84 depletion impairs LSCs’ function and inhibits the development of aggressive and drug-resistant subtypes of AML. At the same time, the expression level of GPR84 was significantly related to the survival time of AML patients.^171,172^ Mechanistically, GPR84 overexpression induces activation of the β-catenin transcriptional cofactors Tcf7l2 and c-Fos as well as genomes associated with Wnt signaling.^173^ GPR84 antagonists have been evaluated in clinical trials to treat ulcerative colitis, idiopathic pulmonary fibrosis, and nonalcoholic steatohepatitis.^174^GLPG1205 as an antagonist of GPR84 has accomplished clinical a phase II trial which is the potential to treat cancers^175^ as well as Compound 33 with improved potency of GPR84 is possible to become a candidate drug for cancer therapy which needs further clinical research.^174^ On the other hand, CRC cells downregulate the expression of GPR84 in bone marrow-derived monocytes/macrophages (BMMs), thereby promoting osteoclastogenesis in an IL-11-dependent manner. Therefore, GPR84 may be a potential therapeutic target to alleviate bone destruction caused by CRC metastasis. Additionally, GPR84 is involved in immune regulation as its expression has been described predominantly in immune cells.^176^ A study proved that GPR84 enhanced macrophage phagocytosis of adipocyte plasma membrane-associated protein (APMAP)-deficient cancer cells.^177^ This means that CPR84 may be a key point linking the tumor metabolic microenvironment with anti-tumor immunity. LY-237 and 6-octylaminouracil have been reported to act as an agonist at GPR84 which is currently in preclinical research.^178,179^ Given the dual role of GPR84 in cancer, it would be meaningful to explore its specific mechanisms in different types of cancer.

##### GPR40

GPR40 is overexpressed in breast tumors as a long-chain fatty acid receptor. One of its ligands, oleic acid (OA) has been shown to participate in cancer cell proliferation.^180^ Exogenous supplement of oleic acid enhanced the fluidity of the plasma membrane and promoted the invasion and migration of HCC cells.^181^ GPR40 overexpression contributed to the oleate-induced proliferation of cancer cells. Using RNA interference, when the GPR40 gene was silenced, oleate-induced proliferation of cancer cells decreased.^182^ In addition, compared with normal ovaries, GPR40 expression is significantly increased in high-grade carcinoma and is higher in in advanced stage disease. It was also found that GPR40 was overexpressed in prostate cancer (PCa) tissue compared with benign prostatic hyperplasia tissue, and OA promoted the aggressive phenotype of PCa cells through FFA1/GPR40, calcium and PI3K/Akt signaling.^183^ These findings suggest that GPR40 plays a cancer-promoting role. The GPR40 antagonist, GW100 resulted in growth inhibition in EOC cell line which means GPR40 is a potential target for cancer therapy.^184^ In addition, there is another small molecule antagonist DC260126 that targeted GPR40, but its application in cancer has not been reported yet.^185^

##### GPR120

GPR120 (encoded by FFAR4 gene) is a receptor for long-chain fatty acids, activated by ω-3 polyunsaturated fatty acids (PUFAs), and expressed in many cell types.^186^ GPR120 was overexpressed in breast cancer cells and was important for the acquisition of chemoresistance. GPR120 enhanced the de novo synthesis of fatty acids that served as GPR120 ligands to activate GPR120 signaling via a feedback mechanism in breast cancer cells. GPR120 antagonist AH7614 or GPR120-siRNA significantly compromised chemoresistance.^187,188^ Also, GPR120 was found to promote tumor cell migration and invasion in pancreatic cancer, colorectal carcinoma(CRC) and bone cancer.^189,190^ However, it has also been shown that GPR120 on M2 macrophages may be beneficial for DHA-mediated anti-prostate cancer effects.^191^ At the same time, the loss of GPR120 in the intestinal epithelial cell leads to increased intestinal permeability, microbiota translocation, and dysbiosis, implying that GPR120 receptors are critical for maintaining mucosal barrier integrity and preventing CRC development.^186^ And other studies have found that it acted as a tumor suppressor because it can suppress tumor cell migration and invasion in melanoma and lung cancer.^189^ Recent studies have developed potent and selective GPR120 agonists such as GW9508,^190^ TUG-891^192^ and compound 39 (a benzofuran propanoic acid analog),^193^ which are expected to become a new kind of anti-cancer drug. A new study has found that GPR120 recognizes single and double bonds in fatty acids and induces different downstream signaling pathway transduction mechanisms, which provides a theoretical basis and structural basis for the development of new high-efficiency unsaturated fatty acid drugs that precisely target GPR120,^194^ and this founding may explain the dual role of GPR120 in cancers.

##### GPR78

In breast cancer, inhibition of GRP78 decreases mitochondrial transport of fatty acids. Thus, fatty acid oxidation is attenuated, leading to the accumulation of essential polyunsaturated fatty acids in the intracellular space. Changes in intracellular fatty acids further increased the serum level of monocyte chemotactic protein 1 (MCP-1) and reduced the expression of the self-recognizing identifier CD47 in the tumor. In addition, inhibition of GPR78 can enhance macrophage infiltration, suggesting a potential link between fatty acid metabolism and cancer immunity,^195^ which means GPR78 may serve as a potential drug target against metastatic human lung cancer. Currently, an antibody against the COOH-terminal domain of GPR78 (anti-CTD antibody) can downregulate pro-proliferative signaling and upregulate p53 in prostate cancer cells and melanoma cells,^196^ and GPR78 protein expression was significantly regulated by miR-936 in laryngeal squamous cell carcinoma (LSCC) cells,^197^ suggesting two possible approaches to target GPR78, but no relevant clinical trials have yet been conducted.

#### Signal transduction of fatty acid derivatives

ATP produced by fatty acid oxidation plays an important role in cell bioenergetics, Src kinase auto-phosphorylation and signal transduction.^198^ ROS produced by fatty acids oxidation of mitochondria contributes to TGF-β, which induces EMT and invasiveness of A549 cancer cells.^199^ But specific metabolite sensing mechanism of ATP and ROS needs further study.

Other lipids derived from fatty acids also have signaling effects that affect metastasis. Sometimes it is through indirect cancer cell autonomic mechanisms. For example, sphingosine-1-phosphate (S1P) is exported from endothelial cells to the circulatory system via the transport protein SPNS2. Knockout of SPNS2 can disrupt the movement of white blood cells from lymphoid tissues into the blood, which paradoxically leads to an increase in the ratio of anti-tumor effect T cells to immunosuppressive T cells, and enhances the inhibitory effect on metastasis.^200^ Fatty acid metabolism and its signal transduction pathways may produce attractive targets for inhibiting metastasis, which is also one of the directions for developing targeted drugs. Prednisone’s bacterial side chain cleavage product 1,4-androstadiene-3,11,17-trione can interact with androgen receptors to contribute to the expression and function of downstream targets, showing pro-proliferation function in prostate cancer cells, but the specific proliferative mechanism needs further study.^201^ Urolithin A destroys the expression and function of Rac family small GTPase 1 (Rac1) and protein activated kinase 1 (Pak1). Subsequently, it also destroys actin depolymerization and migration.^202^ The ability of cancer cells to migrate and invade requires a recombinant actin cytoskeleton,^203^ while Rac1 is the main regulator of the actin cytoskeleton.^204^ Many other studies have also shown that Rac1 is overexpressed in many cancers, and the loss of Rac1 activity inhibits tumor growth.^205,206^ In addition, Uro-A can lead to a dose-dependent anti-cloning effect by increasing the aging-related β-galactosidase activity.^207^ Therefore, the application of urolithin A in cancer prevention or adjuvant therapy is promising.

Overall, GPCRs are essential in fatty acid-related metabolite sensing mechanisms as the main receptor of fatty acids. The concentrations of metabolites are converted into a series of chemical signals which affect the carcinogenesis and metastasis of cancer through binding with their specific receptors. Interestingly, metabolite receptors’ expression levels in cancers are also different from normal cells, which can affect the phenotype of cancers. These mechanisms explain well how changes in fatty acid concentration affect cancer processes. At the same time, these signal transduction pathways and the differential expression levels of metabolite receptors also provide a direction for the development of cancer-specific targeted drugs.

### The function of fatty acid and its metabolites in epigenetics regulation

#### Fatty acids indirectly affect enzymes related to epigenetics

##### Short-chain fatty acid

Short-chain fatty acids (SCFAs) mainly include acetic acid, propionic acid, butyric acid, valeric acid, isobutyric acid, isovaleric acid, caproic acid, etc.^208^ It can be produced by intestinal microbiota and its types and quantity mainly depend on the composition of intestinal microbiota and the consumption of dietary fiber.^209^ SCFAs is a natural inhibitors of histone deacetylase (HDAC) which allows gene transcription, butyrate is the most effective ligand while acetate is the least effective one.^114,119,210–212^ SCFAs regulate epigenetics mainly by changing the function of HDAC. HDAC inhibition causes histone acetylation to produce negatively charged histones. When they interact with negatively charged DNA, the chromatin structure is loosed, producing a transcriptionally active conformation that upregulates tumor suppressor genes of CRC in epigenetics.^213^ And butyrate can induce a four-fold increase in the transcription level of TNF receptor superfamily member 25 (Tnfrsf25) and death associated protein kinase 1 (Dapk1) (apoptosis-related genes), the increased expression of these genes may be due to the reverse effect of butyrate on histone deacetylation^214^ as the promoter regions of Dapk1 and Tnfrsf25 are close to the site where histone modification occurs.^215^ Meanwhile, HDAC inhibition can also inhibit or trigger the expression of miRNA in some cancer samples.^216^ For example, HDAC inhibition can trigger the expression of miR-15a and miR-16 in CLL, which expression is usually reduced in cancer.^217^ Acetylation can also promote the activation, nuclear translocation and DNA binding of transcription factors (such as STAT3, NF-kB, FoxP3, N-FAT and RUNX1).^118,218,219^ In addition, sodium propionate (SP) affects the expression of survivin and p21, inducing cell cycle arrest especially in the G2/M phase, and then leading to apoptosis that can be applied to the targeted treatment of lung cancer.^220^ Additionally, SP can upregulate the surface expression of MHC class I related chain A/B(MICA/B) which is NKG2-D type II integral membrane protein (NKG2D) ligand by increasing overall acetylation and propionylation and then inhibiting lysine acetyltransferase (KAT), which is not independent on GPR41/GPR43 receptors, but functional mitochondria.^221^ There are already some HDAC inhibitors, such as SAHA (Vorinostat),^222–224^ entinostat,^225^ valproate^226^ and romidepsin.^227^ Romidepsin and Vorinostat have been approved for the treatment of T-cell lymphoma by the FDA,^228,229^ but current research has not shown that they can be used as colorectal cancer treatment drugs. There are currently several phase I clinical trials exploring the combination regimen of HDAC inhibitors to achieve better cancer treatment^230,231^ and many novel HDAC inhibitors are undergoing phase I and II clinical trials.^232,233^ In addition, SCFAs may directly affect the transcription of HDAC genes.^118^ In GPR43-deficient mice, cycle adenosine monophosphate (cAMP)-protein kinase A (PKA)-cAMP response element binding (CREB) pathway is enhanced, resulting in the overexpression of HDAC. Similarly, more neutrophils infiltrated into tumors and the colon lamina propria in GPR43-deficient mice. In addition, butyric acid inhibits HDAC expression and methylation of inflammation inhibitory factors through GPR43. From this point of view, SCFAs are a kind of epigenetic inhibitor, which play a pivotal role in multiple stages of colon cancer.^159^

##### Medium-chain fatty acids

In addition to SCFAs, it is worth noting that the latest research shows that the addition of medium-chain fatty acids C8 or C10 to glioblastoma cells can affect the citric acid cycle, affecting the Warburg effect, glutamine/glutamate metabolism and ketone body metabolism. C8 leads to an increased ketone body production while C10 mainly affects the cytoplasmic pathway by stimulating fatty acid synthesis.^234,235^ Also, medium-chain fatty acids, like Valproic acid or valproate (VPA) inhibit HDAC, thus exerting similar anti-cancer effects to SCFAs in regulating epigenetics.^236^

##### Acetone body

Acetone body can be used as both an energy substrate and signaling molecule. Nuclear receptor peroxisome proliferator-activated receptor alpha (PPARα) is one of the main regulators of ketogenic effects, which can integrate nutritional signals into the transcriptional network that regulates the activation of fatty acid β oxidation and ketogenic effects. β-hydroxybutyrate (3-OHB), a kind of ketone body, has been identified as a class I HDAC inhibitor, which establishes a link between liver lipid metabolites and epigenetics. 3-OHB binds to specific hydroxyl-carboxylic acid receptors, and then inhibits HDACs, FFARs, and the NOD-like receptor protein 3 inflammasome, resulting in the inhibition of lipolysis, inflammation, oxidative stress, cancer growth and angiogenesis.^237^ Clinical research finds that the ketogenic diet with chemotherapy can improve the survival rate of patients with advanced local or metastatic breast cancer.^238^ This evidence suggests that there is a close link between ketone bodies and tumor epigenetic regulation, and targeting ketone bodies may provide new ideas for cancer treatments. In addition to interfering with ketone body metabolism through diet, enzymes in the ketone body metabolic pathway may also be targets, such as 3-oxoacid CoA-transferase 1 (OXCT1) which can catalyze the first step and limit the rate of ketone body metabolism step. Studies have shown that the expression of OXCT1 is significantly increased in different types of cancer cells and provided great potential in cancer therapy.^239^

##### ω-3polyunsaturated fatty acid (ω-3PUFA)

The epigenetic regulation of ω-3PUFA may be directly attributed to their electrophilic oxidized derivatives, which are produced through endogenous enzymatic or non-enzymatic pathways.^240–245^ Electrophilic lipids act as epigenetic modifiers by directly adding to histones, regulating catalytic histone modification or DNA methylation of enzymes, and controlling miRNA expression.^246–250^ ω-3PUFA can modulate epigenetic events to regulate cellular processes associated with carcinogeneses, such as proliferation, differentiation, inflammation, and angiogenesis,^251^ which is expected to be an attractive dietary supplement to improve the prognosis of cancer patients.^252^ The treatment of docosahexaenoic acid (DHA) and eicosapentaenoic acid (EPA) can downregulate the expression of EZH2, which is the core component of polycomb repressive complex (PRC2), and PRC2 can lead to H3K27me3 modification^253^ in vitro. On the other hand, EZH2 is closely associated with miRNA silencing. It can bind to the miRNA promoter and regulate the expression of miRNA and finally regulating epigenetics.^216^ Fish oil rich in EPA and DHA can reduce breast cancer metastasis in mouse breast cancer models and reduce both the migration and the invasion of cancer cells by down-regulating the expression of CD44 in vitro. CD44 is a kind of cell surface protein that can combine matrix collagen and MMP-9 promoting extracellular matrix(ECM) remodeling.^254^ Preclinical and clinical trials evaluating the pharmacokinetics, efficacy, and drug-related toxicity of CD44 monoclonal antibodies have been conducted in tumors with CD44 expression.^255^ DHA also directly blocks the MMP-9 expression by inhibiting the PPARγ/NF-κB pathway.^256^ In addition, DHA can induce Sirtuin 1 (SIRT-1) expression in a variety of cells including colonic epithelial cells,^257,258^ an NAD^+^ dependent histone deacetylase, which mainly targets histones and non-histone proteins including transcription factors. The combination of fish oil (FO) and pectin (butyrate) can enhance the apoptosis of colon cells, which is related to the expression of genes involved in apoptosis.^259–261^ A study has found that dietary supplementation of pectin significantly increased the abundance of butyric acid bacteria in the gut, promoting butyric acid production, and enhanced the therapeutic effect of anti-PD-1 mAb, which establish a connection between metabolism and immune response.^262^ The combination of DHA and butyric acid significantly reduced the methylation of apoptosis related-genes promoters, indicating that the induction of apoptosis by DHA and butyric acid is partially mediated by changes in the methylation status of apoptosis-related genes.^263^

Non-coding RNA-mediated regulation of gene silencing is another mechanism of epigenetic regulation of ω-3PUFA, which may interfere with gene expression. Carcinogenic targets of several miRNAs (miRNA-19b, miRNA-26b, and miRNA-203) that are down-regulated by the FO plus pectin diet.^264^ ω-3PUFA downregulates miRNA-26 in cholangiocarcinoma cells, and miRNA-26a/b targets 15-hydroxyPGdehydrogenase (15-PGDH) mRNA and inhibits its translation. 15-PGDH can catalyze the oxidation of the 15 (S) -hydroxyl group of prostaglandin E2 (PGE2) which is a pro-inflammatory lipid medium promoting carcinogenesis, so the downregulation of miRNA-26 may be related to the ability of ω-3PUFA to inhibit cancers.^265^ Interestingly, ω-3PUFA also directly upregulates 15-PGDH which acts as a tumor suppressor in lung and colon cancer.^266,267^ Several compounds that can induce 15-PGDH expression have been reported, including histone deacetylase inhibitors, nonsteroidal anti-inflammatory drugs, and peroxisome proliferator-activated receptor-γ agonists. Therefore, 15-PGDH can be considered a novel molecular target for cancer chemoprevention and therapy.^268^ In addition to affecting miRNA expression, ω-3PUFA also affects its secretion, such as DHA inhibits angiogenesis by triggering exosome secretion of miRNAs which promotes the expression of angiogenic genes in endothelial cells.^269^ So, taking full advantage of the anti-cancer effect while inhibiting its cancer-promoting effect of omega-3PUFA may lead to better therapy in cancers, all of which are the main epigenetic modification, and well-known epigenetic markers.^15^ Several phase I and phase II clinical trials of omega-3PUFA supplementation in solid tumors have been completed, however, the outcome is ambiguous and requires more precise evidence.

##### Fatty acid ester of phloridzin

Fatty acid esters of phloridzin significantly inhibit the growth of human hepatocyte HepG2 cells, human breast adenocarcinoma cells, and acute monocyte leukemia cells. It inhibits DNA topoisomerase IIα activity, induces cell cycle arrest in G0/G1 phase and apoptosis by activating caspase-3 and reducing ATP levels and mitochondrial membrane potential in HepG2 cells. The antiproliferative effect of phloridzin DHA ester may be related to the anti-apoptotic gene (BCL2), growth factor receptor (EGFR family, IGF1R/IGF2, PDGFR), and its downstream signaling partners (PI3K/AKT/mTOR, Ras/Raf/MAPK), cell cycle mechanisms like cyclin-dependent kinases (CDK), topoisomerase IIα and IIβ (TOP2A, TOP2B) and down-regulation of epigenetic regulators (HDAC).^270^ HDAC plays important role in histone modification, which is also a crucial part of epigenetic regulation in cancer.^271^ HDAC inhibitors (HDACi) for cancer treatment are approved by the FDA,^272^ which are functional by blocking the catalytic sites of HDACs and changing cellular acetylation patterns, causing the death of cancer cells.^273^

##### Other fatty acid derivatives

Resveratrol (3,4,5-trans-trihydroxystilbene) is a plant antitoxin produced in various plants (such as grapes, peanuts, and cranberries) due to pathogen invasion or other environmental stresses.^274^ DMU-214 is a kind of metabolite of cytotoxic resveratrol analog DMU-212, which can regulate several genes related to migration and proliferation (SMAD7, THBS1, IGFBP3, KLF4, Il6, ILA, SOX4, IL15, SRF, RGCC, GPR56) and protein (GPR56, RGCC, SRF, SMAD7, THBS1)^275^ to reduce cell proliferation and movement. DMU-214 inhibition of GPR56 expression, the decrease of GPR56 expression triggered by DMU-214 was accompanied by the inhibition of SKOV-3 cell motility.^276^ The ginseng metabolite protopanaxadiol (PPD) can induce the expression of BH3-only proteins Puma and Noxa, thereby promoting the death of colorectal cancer cells, and also enhancing the anti-cancer effect of fluorouracil (5-FU). In addition, PPD also induces the expression of autophagy and Bcl2 family apoptosis regulator myeloid cell leukemia-1(MCL-1) that can significantly increase PPD-induced cell death. Interestingly, PPD inhibited the expression of fatty acid and cholesterol biosynthesis-related genes, and induced cancer cell death with fatty acid synthase inhibitor cerulenin.^277^ There is no doubt that these metabolites seem to provide an alternative direction for cancer treatment. However, these metabolites regulate gene expression in which ways that needs to be further investigated.

#### Fatty acids directly affect epigenetics through substrates

##### Acetyl-CoA

Acetyl-CoA establishes an important link between energy metabolism and chromatin regulation.^278,279^ Fatty acids can affect the level of acetyl-CoA and then directly influence histone acetylation which plays a crucial role in metabolism, signal transduction, and epigenetics. On the one hand, ubiquitous de novo fatty acid synthesis in cancer cells needs to use acetyl-CoA which is provided by the same acetyl-CoA reservoir of histone acetylation. The acetyl-CoA pool is provided by ATP citrate lyase (ACLY) and acetyl-CoA synthase (ACSS). ACLY cleaves citrate into oxaloacetate and acetyl-CoA, ACLY is essential for tumorigenesis in mouse cancer models, and its compound inhibitors with high IC50 values have antitumor efficacy in xenograft models of lung and prostate cancer. However, no active ACLY inhibitors have been reported in vivo tumor models.^106^ ACSS links acetate and CoA to produce acetyl-CoA,^280^ playing an important role in acetate-dependent tumors. Lacking ACSS2 attenuates tumor burden without any phenotypic defects. Therefore, many ACSS2 inhibitors are needed to be tested in tumor models.^281,282^ MTB-9655, a kind of small molecule inhibitor of ACSS2, is in phase 1 clinical trial as a potential treatment for patients with cancer (NCT04990739). Reducing the expression of acetyl-CoA carboxylase 1 (ACC1) increases the global histone acetylation and gene expression by reducing FA synthesis,^283^ and low-potency ACC1 inhibitor TOFA resulted in tumor regression in MYC-induced renal tumors,^284^ at the same time, ND-646, a nanomolar inhibitor of ACC1, inhibits tumor fatty acid synthesis and tumor growth of lung cancer in vivo.^285,286^ However, no clinical trials about them have been conducted. On the other hand, fatty acid oxidation increases acetyl-CoA levels, leading to an increase in histone acetylation^287^ and the expression of lipase and acyl-CoA short-chain synthetase family member 2 (ACSS2). Acetate promotes the expression of lipase and acyl-CoA short-chain synthetase family member 2 (ACSS2). In T cells lacking glucose, acetate promotes histone acetylation, increases IFN-γ production, and promotes tumor clearance.^288^ There is evidence that increased histone acetylation can drive increased expression of the transcription factor Twist2 that induces EMT.^289^ Overall, inhibiting the fatty acid synthesis and increasing acetyl-CoA levels can promote global histone acetylation which is a potent development prospect for targeted therapy in cancers.

##### Organic acid

**Fig. 3:**
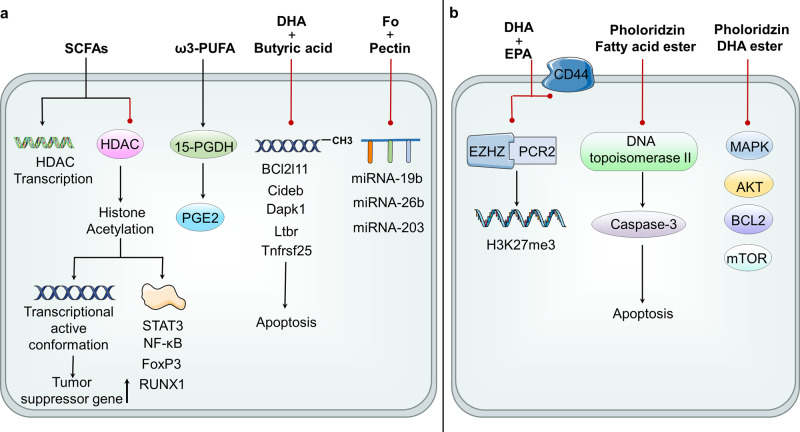
Epigenetic regulation of fatty acids and their intermediate metabolites in cancer. ACLY cleaves citrate into oxaloacetate and acetyl-CoA, while ACSS connects acetate and CoA to generate acetyl-CoA and generates acetylcholine depot, which together with organic acids affects histone posttranslational modifications and induces Twist2 expression increase. **a** CRC: SCFAs and ketone bodies inhibit HDAC enzyme to make histone acetylation, up-regulate the tumor suppressor gene, and promote the activation of transcription factors STAT3, NF-kB, FoxP3, N-FAT and RUNX1. It can also directly inhibit the transcription of HDAC gene. ω-3PUFA up-regulates 15 PGDH and catalyzes the oxidation of the 15 (S) -hydroxyl group of prostaglandin E2 (PGE2), fish oil and pectin reduce the pro-apoptotic Bcl2l11, Cideb, Dapk1, Ltbr and Tnfrsf25 promoter methylation which promotes cell apoptosis, and down-regulation of miRNA (miRNA-19b, miRNA-26b and miRNA-203). **b** BCC: DHA and EPA down-regulate the expression of EZH2, and then H3K27me3 modification. Fish oil can down-regulate the expression of CD44, promote ECM remodeling, and inhibit the migration and invasion of cancer cells. DHA inhibits the PPARγ/NF-κB pathway, blocking MMP-9 expression, and triggers exosome secretion of microRNAs that promote angiogenesis genes to inhibit angiogenesis; phloridzin fatty acid esters inhibit DNA topoisomerase IIa activity and activate caspase, inducing apoptosis, Phloridzin’s DHA ester down-regulates BCL2, growth factor receptor and PI3k/AKT/mTOR, Ras/Raf/MAPK and HDAC

Histones are regulated by many posttranslational modifications (PTMs)^290^ and then regulate gene expression at the epigenetic level. There are two specific mechanisms: First, histone PTM changes the net charge of histone molecules or changes the interaction with nucleosomes to adjust the packaging of chromatin directly. Second, histone PTM recruits PTM-specific binding proteins to regulate the structure and function of chromatin.^291,292^ Histone PTMs play a crucial role in various biological processes such as cell proliferation and differentiation. An abnormal histone modification will cause cancers and other diseases.^293,294^ In addition to acetyl groups, histone PTM can also reversibly add and cleave other acyl groups (formyl, propionyl, butyryl), myristoyl groups, and dicarboxylic acids (malonate, succinate, pentane).^295–299^ The studies focus on three kinds of histone modifications: myristoylation of N-terminal glycine residues (N-myristoylation),^300^ and the crotonylation (Kcr) form of lysine residues.^298^ The fatty acid acylation mechanism of histones directly links fatty acid levels to histone epigenetics (Fig. 3), but the specific mechanisms of various organic acids regulating epigenetics and their roles in diseases need to be further investigated. Abnormal epigenetic histone modifications are related to cancer pathogenesis, especially in prognosis and invasiveness of care, which may lead to clinical outcomes.^301^ For example, the prognosis in breast cancer is associated with the reduction of lysine acetylation (H3K9ac, H3K18ac, H4K12ac), methylation (H3K4me2, H4K20me3) and arginine methylation (H4R3me2).^302^

Fatty acids and their derivatives can not only affect the concentration of substrates related to epigenetics but also affect the activities of enzymes related to epigenetics, thereby regulating the expression of genes in epigenetics. This effect is bilateral and can produce both cancer-promoting and cancer-suppressing effects. Whatever, it provides us with a direction: changes in metabolite concentration can affect the level of gene expression, which may be a great way to prevent and cure cancers, even though there is still a long way to go.

Cancer cells show higher metabolic flexibility in responding to metabolic reprogramming, this implies that we are able to increase the intake or production of certain tumor suppressor metabolites to inhibit the process of carcinogenesis and metastasis of cancer. Based on this point of view, many studies have been carried out, such as exogenous intake of fatty acids or intestinal probiotics that produce short-chain fatty acids,^303,304^ which will be expected to provide powerful methods for tumor prevention and treatment in the future.

## Metabolite sensing and epigenetic regulation of protein metabolism

### Signal transduction of protein metabolism

Mammalian target of rapamycin (mTOR)-mediated amino acid sensing is an important metabolite sensing mechanism for amino acids. mTOR itself is a protein kinase. It can detect the abundance of amino acids by working with specific sensor proteins, which mainly are the followings: lysosomal proteins slc38a9 (uncharacterized human member 9 of the solute carrier family 38)^305^ and cas-tor1^13^ are proven to be metabolite sensor of arginine, Sestrin2 is the leucine sensor,^14^ while methyl donor S-adenosylmethionine target of rapamycin (SAMTOR) is a methionine receptor.^306^ Meanwhile, a study suggested that map4ke (mitogen-activated protein kinase-3) could also act as an upstream amino acid receptor of mTOR.^307^ Leucine synthetase and vacuolar H+-ATPase are two intracellular amino acid sensing elements that are closely related to the activation of mTOR1. When leucine concentration increase, leucine synthase migrates to the lysosome. In lysosomes, leucine synthetase facilitates the proper nucleotide loading of the Rag GTPase heterodimer complex, which stimulates the activity of mTORC1 through the mechanism that will be described later.^308^ Vacuolar H+-ATPase is located in the lysosomal membrane and has been identified as a key component of the lysosomal surface amino acid sensing mechanism. It seems to be very sensitive to the accumulation of amino acids in lysosomes. By directly interacting with the regulator-rag complex, it stimulates the activation of mTORC1 when the concentration of amino acids in the lysosome cavity increases.^309^

When the cell senses the presence of amino acids, it activates mTORC1. Amino acids recruit mTORC1 on the surface of the lysosome, where the activator Ras (rat sarcoma) homolog enriched in the brain (Rheb), the direct upstream activator of mTORC1, is located.^309,310^ This recruitment of mTORC1 on the lysosomal membrane is mediated by Rag GTPase heterodimers.^311,312^ The specific mechanism is that the increased amino acid availability stimulates the transfer of GDP and GTP loading of the Rag heterodimer,^311^ which converts Rag GTPase to the active configuration phase,^313^ thereby promoting Rag heterodimer to bind to mTORC1 that recruits to the lysosomal surface.^311^ Then the “docking” of mTORC1 with lysosomes requires a protein complex called regulators, which provides an anchoring mechanism between the Rag GTPase heterodimer bound to mTORC1 and the lysosomal membrane. In this way, it allows the activation of mTORC1.^310^ Interestingly, the study found that when the amino acid adequacy is low, a protein subcomplex called GTPase-activating target of rapamycin 1(GATOR1) has been shown to inactivate the rag heterodimer complex, thereby inhibiting the ability of RAG GTPase heterodimer to recruit mTORC1 on the surface of the enzyme body.^314^ When activated, mTOR will phosphorylated p70-S6 kinase 1 (S6K1) and 4E binding protein 1(4E-BP1)), and the phosphorylated S6K1 and 4E-BP1 activate the protein translation initiation complex, then promote protein synthesis. Specially, when 4E-BP1 is phosphorylated, it will promote the release of eukaryotic initiation factor 4E (eIF4E). Therefore, eIF4E is allowed to combine with eIF4G and eIF4A to form the eIF4F translation initiation complex, then activating protein synthesis.^315^ At the same time, mTOR can also play a broad regulatory role by phosphorylating other effector proteins.^315^ Surprisingly, new research found that glutamine and asparagine activate mTORC1 through a Rag GTPase-independent mechanism that requires ADP-ribosylation factor 1 (Arf1), while leucine, arginine, and methionine signal to mTORC1 through the well-characterized Rag GTPase signaling pathway as we mentioned before. This research shows mTORC1 is differentially regulated by amino acids through two distinct pathways.^316^

Amino acid sensing mediated by mTOR plays an important role in the pathogenesis of tumors. Of all 97 human cancers, mTORC1 is considered to be up-regulated by approximately 70%.^121^ It has newly been reported to relate to acute myeloid leukemia,^317^ subependymal giant cell astrocytoma,^318^ triple-negative breast cancer^319^ and pancreatic tumor.^320^ mTOR has also been found to be used in the treatment of cancers. So far, two generations of mTOR inhibitors have been developed and have shown promising tumor suppression in preclinical studies. The clinical application of mTOR inhibitors has been successfully tested in treating advanced renal cell carcinoma, neuroendocrine tumors, and HER2-positive breast cancer.^121^ It can also ablate cisplatin-resistant salivary gland cancer stem cells, especially combined with platinum-based chemotherapy.^321^ Meanwhile, a new study also found that enhance mTOR-targeted cancer therapy can be enhanced by combining of other drugs such as mitoxantrone, an inhibitor of the eukaryotic elongation factor-2 kinase, which is overexpressed in cancer cells and is required for the survival of stressed cells.^322^

#### The amino acid sensing related to G protein-coupled receptor

G protein-coupled receptors are an important metabolite-sensing receptor binder antagonist, and most GPCRs bind to short-chain fatty acids.^118^ However, there are a few GPCRs that combine with amino acids and their metabolites, such as tryptophan metabolites nicotinic acid and Kynurenic acid (the host’s intermediate for tryptophan metabolism), and indole-3-aldehyde (derived from tryptophan Bacterial metabolism).^117^ Their main function is mainly about adaptive immunity and intestinal barrier that regulates inflammation. GPR109A combines not only with butyrate of short-chain fatty acids but also with niacin, a metabolite of tryptophan.^323^ Under physiological conditions, GPR109A can only be activated when the concentration of butyrate is sufficient, and the concentration of niacin is insufficient to activate GPR109A.^138^ At the same time, some studies have found that after activating GPR109A by niacin, it can reduce the secretion of proinflammatory cytokines made by macrophages, monocytes and epithelial cells.^324^ Meanwhile, recently study found that GPR109A-AKT signaling pathway was related to the butyrate markedly inhibited glucose transport and glycolysis of colorectal cancer cells.^325^ Also, another experience found that supplementation of ketogenic diet with a pharmacological antagonist of the 3HB receptor GPR109A in rats abolished the antitumor effects.^326^ Although currently, the researches related to GPR109A’s are mainly focused on its role in inhibiting inflammatory mediators, one research found that it can promote the occurrence of ApcMin/+-driven colon cancer through animal experiments. Adenomatous polyposis (APC) gene mutation is one of the inherited forms of human colon cancer. IL-10 and Treg cells are suppressed after the mutation, and IL-17 promotes Apcmin/mouse (the mouse who has an express point mutation in one copy of APC gene) to develop a colon cancer. However, as for how GPR109A promotes the occurrence of ApcMin/+ driven colon cancer, its specific mechanism needs further study.^115^ It is found that colon cancer cells were more sensitive to the depletion of tryptophan compared with normal human colonic epithelial cells.^327^

Studies have also found that D-tryptophan and the essential amino acid D-phenylalanine can be combined with GPR109B. GPR109B, like GPR109A, also plays an important role in the immune-free system, but its specific role in tumors is currently unknown.^328^ More progress studies of GPR109A in cancer can be seen in the previous fatty acid portion. GPR35 is also related to amino acid metabolites kynurenic acid, lysophosphatidic acid and pamoic acid. It is mainly a GPR that is poorly expressed in the gastrointestinal tract, especially in the small intestine and colon.^329^ But its main role is to focus on the onset of colitis, and no research related to malignant tumors has been seen. Study shows the over-expression of GPR137 is associated with the growth of tumor cells, while under-expression of GPR137 has been shown to inhibit cell proliferation in several different types of cancers, but its role in amino acid sensing is still unclear.^330^

There are also another kind of GPRs which was combined with protein that related to the tumor development, GPR56. GPR56 binds transglutaminase 2 to suppress tumor metastasis^331^ and binds collagen III to regulate cortical development and lamination.^332^ GPR56 has shown a high expression level in many cancers, such as esophageal cancer,^333^ glioblastoma,^334^ human melanoma^335^ and colon cancer^276^ as a cancer promoting factor which is different from GPR109A and GPR43. Its cancer-promoting effects are related to proliferation, migration, angiogenesis, cell adhesion, apoptosis and cell cycle regulation.^335–337^ The expression and activation of GPR56 can regulate the progression of melanoma by specifically inducing IL-6 production after the dissociation of the N-terminal fragment and the self-activation of the C-terminal fragment, thereby promoting cell migration and invasion.^335^ The higher level of GPR56 expression in colon cancer patients means a poorer prognosis. The depletion of GPR56 significantly inhibits cell proliferation, migration and invasion. Further research shows that GPR56 overexpression accelerates epithelial-mesenchymal transition by activating PI3K/AKT signaling, promoting colorectal cancer (CRC) cell metastasis.^276^ However, GPR56 could inhibit tumor growth and metastasis.^338^ Melanoma angiogenesis is inhibited as GPR56 prevents melanoma cells from producing VEGF. It is important in cancer metastasis due to its antagonistic effect with melanoma.^337^ There were studies related GPCR to amino acid. Leucine-rich repeat-containing G-protein coupled receptor 5 (LGR5) is highly expressed in cancer stem cells. The loss of LGR5 in colon cancer cells enhanced resistance to irinotecan and 5-fluorouracil and increased expression of adhesion GPR56, thus the knockdown of GPR56 in multiple colon cancer cell lines led to suppression of tumor growth and decreased drug resistance, which maybe a new target drug for colon cancer.^339^ Considering its dual role, developing target drugs against it is significant. There are already several functional antibodies against GPR56, but they have only been shown to have pharmacological and in vitro activity, and studies in vivo are still lacking, which indicates that functional antibodies are valuable tools for GPCR research.^340–342^

#### The amino acid sensing related to glutamic acid

Glutamic acid, the first metabolite of glutamine, also plays an important role in the development of tumors. It also related to amino acid sensing. Glutamic acid produced by glutamine hydrolysis can be exported from tumor cells through transporters such as recombinant solute carrier family 7, member 11 (SLC7A11 or xCT). Glutamate plays an important role as a signaling molecule in regulating cancer metastasis. The destruction of glutamate cysteine anti-transporter xCT (also known as slc7a11) leads to a retention of cellular glutamate, which makes glutamate fail to function out of the cells, resulting in reduced proliferation and reduced invasion of non-small cell lung cancer.^343^ When xCT is functional and glutamate is exported, glutamate can function through multiple types of receptor signals.^344^ It can activate MAPK and AKT signaling that promote tumorigenesis by combining with ionic and metabolic glutamate receptors α-amino-3-hydroxy-5-methyl-4-isoxazole-propionic acid receptor (AMPAR) and gene expression of metabolic glutamate (GRM), thereby reducing intercellular adhesion and increasing tumor erosion. Meanwhile, activity of hypoxia inducible factor-1 (HIF-1), a factor that plays an important role in glycolysis of tumor under hypoxia conditions, is significantly increased by glutamine metabolism.^345^

Speak of treatment, many studies have shown that inhibiting xCT has the effect of inhibiting tumor growth,^346,347^ maybe the potential therapeutic target for tumor.^347–349^ Importantly, a recent study showed that xCT inhibition works synergistically with the non-therapeutic agent anti-CTLA-4150. This study laid the foundation for the clinical application of specific xCT inhibitors to expand the efficacy of existing anti-cancer immunotherapy.^350^

At present, there is also progress in the research of tumor therapy about glutamic acid. On the one hand, the biological effects of glutamate can be reduced by inhibiting glutamate receptors. Studies have found that reducing gene expression of metabolic glutamate receptor 1 (grm1) through genetic manipulation can proliferate the ER-positive breast cancer cells.^351^ In addition, treatment of metabolic glutamate receptor 5 antagonists respectively reduced the metastasis and invasion in vivo and in vitro in oral cancer cell line b88-SDF-1.^352^ On the other hand, tumor growth can be directly inhibited by inhibiting glutamine transport. Glutamine is introduced into cells through various transporters, amino-acid transporter 2 (ASCT2) (also known as slc1a5).^353^ For example, by reducing the expression of ASCT2 to block glutamine uptake, it leads to a decrease in the proliferation of prostate cancer cells, and activates the mTORC1 signal.^41^ Meanwhile, it also reduces the migration of osteosarcoma and triple-negative breast cancer.^354^ Another treatment is the use of new 2-amino-4-bis (aryloxybenzyl) aminobutyric acid (aba) derivative drugs designed to target act2, such as v-9302, which has been proven to reduce cell proliferation, increase cell death and oxidative stress. At the same time, v-9302 can also reduce glutamine uptake by preventing excess glutamine transporters’ function, thereby inhibiting the development of cancer.^355^ Meanwhile, the study found that glutamate metabolism-related products in the urine of prostate cancer patients were significantly up-regulated, which may have clinical significance for the diagnosis of prostate cancer.^356^ L-type amino-acid transporter 1 (LAT1) is also a cancer-associated amino acid transporter gene related to mTOR, which already showed an essential role in the cell proliferation and occurrence of malignant phenotypes.^357^ It is also increased in human ovarian cancer cell lines, thus LAT1 may be a target for combination therapy with anti-proliferative aminopeptidase inhibitors to combat ovarian cancer.^358^

#### The metabolite sensing related to downstream amino acid products

**Fig. 4:**
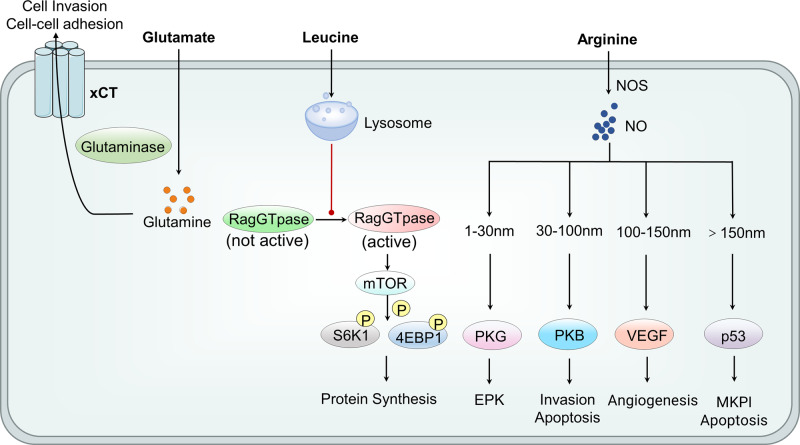
The pathways of metabolite sensing of glutamate, leucine and ariginie. It includes three pathways of metabolite sensing of protein. Glutamic acid produced by glutamine hydrolysis can be exported from tumor cells through transporters such as XCT. When Xct is functional and glutamate is exported, glutamate can function through multiple types of receptor signals, thereby reducing intercellular adhesion and increasing tumor erosion. Leucine concentration increasing leads to a migration from leucine to the lysosome, where it facilitates the proper nucleotide loading of the Rag GTPase heterodimer complex, which stimulates the activity of mTORC1. When activated, mTOR will phosphorylate S6K1 and 4E-BP1, and the phosphorylated S6K1 (phosphorylates p70-S6 kinase 1) and 4E-BP1 (4E binding protein 1) activate the protein translation initiation complex, then promote protein synthesis. Arginine can be transported to NO through NOS in cells. Below 150 nm, NO stimulates the proliferation of tumor; above this concentration, NO promotes cancer cell death: When between 1nm-30nm, NO activates protein kinase G, whose role is to promote angiogenesis and endothelial cell proliferation. Between 30 and 100 nm, NO activates other downstream kinases, such as protein kinase B (PKB or AKT), and stimulates proliferation and anti-apoptotic responses in tumor cells. Between 100 and 150 nm, NO stabilizes hif-1α, thereby increasing the production of vascular endothelial growth factor (VEGF in endothelial cells and cancer cells, leading to angiogenesis of tumor. Above 150 nm, the tumor suppressor gene p53 will be phosphorylated and activated, mitogen-activated protein kinase 1 (mkp1) will be overexpressed, and cellular respiration will be inhibited, leading to tumor cell apoptosis

Nitric oxide (NO) is a metabolic result of arginine catabolism. According to its time, location and concentration, it has the effect of inhibiting tumor and promoting tumor.^359^ It promotes carcinogenesis through a variety of mechanisms, including increasing angiogenesis and limiting the host’s immune response to the tumor. However, it can also be used as a tumor suppressor molecule by activating caspase and inhibiting p53.^359^ The study found that 150 nM is the NO concentration threshold: below this concentration, NO stimulates the proliferation of tumor; above this concentration, NO promotes cancer cell death.^360^ Especially between 1nM-30nM, NO activates protein kinase G, phosphorylated protein kinase G, whose role is to promote angiogenesis and endothelial cell proliferation, including ERK (extracellular signal-regulated kinase).^360^ Between 30 and 100 nM, NO activates other downstream kinases, such as protein kinase b (PKB or AKT), and stimulates proliferation and anti-apoptotic responses in tumor cells.^361^ Between 100 and 150 nM, NO stabilizes HIF-1α, thereby increasing the production of vascular endothelial growth factor (VEGF) in endothelial cells and cancer cells, leading to angiogenesis of tumor.^362,363^ When the concentration of NO exceeds 150 nm, the nitroso stress in the tumor microenvironment increases and the independent stress sensor is activated. In this way, the tumor suppressor gene p53 will be phosphorylated and activated, mitogen-activated protein kinase 1 (MAPK1) will be overexpressed, and cellular respiration will be inhibited, leading to tumor cell apoptosis^363^ (Fig. 4). Besides, 8-Hydroxyquinaldic acid, the end-metabolite of tryptophan, has an evidenced anti-proliferative activity towards cancer cells. It is found that it can induce changes in protein expression of cell cycle regulators (CDK4, CDK6, cyclin D1, cyclin E) and CDKs inhibitors (p21 Waf1/Cip1, p27 Kip1), then inhibit proliferation and mitochondrial activity in colon cancer HT-29 and LS-180 cells, as well as decrease DNA synthesis.^364^ Cadaverine is a downstream product of lysine. Its treatment of breast cancer cell lines corresponding to inhibit cellular movement and invasion, moreover, rendered cells less stem cell-like through reducing mitochondrial oxidation. It is assumed that cadaverine production seems to be a regulator of early breast cancer.^365^ All in all, amino acid metabolism has a large difference between normal cells and tumor cells, and this difference has significant clinical significance. For example, the study found that before and after colorectal cancer surgery, amino acid levels such as glycine and arginine have significant differences, which may be used as potential assessment of metabolites for diagnosis of cancer of colon.^366^ Besides, it’s demonstrated that the PLS-DA model using the six metabolites (glycine, valine, methionine, citrulline, arginine and C16-carnitine) had a strong ability to identify lung cancer.^367^ However, other than the above four pathways, the specific mechanism between these metabolite changes and cancer development needs more research, so as to better provide new tools for clinical diagnosis and treatment (Table 1).

**Table 1:** Signal transduction-mediated metabolites in cancers

Category	Receptor	Metabolites	cancers	Effects mediated by receptor	Ref./refs.
Glucose	GPR31	Lactic acid and pyruvate	Colorectal cancer Prostate cancer	Tumor progression	^34,35^
	SUCNR1	Succinate	Cancers with SDH germline mutation	Cancer metastasis and invasion Angiogenesis Macrophage migration and M2 polarization	^36–40^
Fatty acid	GPR109A	Niacin and butyric acid	Colorectal cancer	Cell apoptosis, against carcinogenesis	^112^
		β-Hydroxybutyrate	Colorectal cancer	Tregs and IL-10-producing T cell activation	^137^
		Short-chain fatty acid β-Hydroxybutyrate	Breast cancer	Apoptosis and cell cycle stagnation	^138^
	GPR109B	3-Hydroxyoctanoate	Breast cancer	Cell proliferation Inhibition of FAO	^136,146,157^
	GPR43	Short-chain fatty acid	Colorectal cancer	Inhibition of recruitment and migration of neutrophils Failure of CD8(+) T cells Excessive activation Of DCs	^160,163^
	GPR84	Medium-chain fatty acid	Acute myeloid leukemia	Activation of Wnt signaling	^173^
			Colorectal cancer	Inhibition of osteoclastogenesis	^176^
			B cell lymphoma	Macrophage phagocytosis	^177^
	GPR40	Oleic acid	Hepatocellular carcinoma	Cell proliferation, migration and invasion	^180,181^
			Ovarian cancer	Cell proliferation, migration and invasion	^182^
			Prostate cancer	Activation of PI3K/Akt signaling Cell invasion	^183^
	GPR120	Long-chain fatty acid	Breast cancer	De novo synthesis of fatty acids	^187,188^
			Pancreatic cancer Colorectal carcinoma Bone cancer	Cell migration and invasion	^189,190^
			Prostate cancer	Activation of M2 macrophages	^191^
			Melanoma	Inhibition of cell migration and invasion	^189^
			Lung cancer		
	GPR78	Unknown	Breast cancer	Mitochondrial transport of fatty acids Inhibition of Macrophage infiltration	^195^
Amino acid	mTOR	Leucine	Acute myeloid leukemia Subependymal giant cell Astrocytomas Triple-negative breast cancer Pancreatic tumor	Activating protein synthesis	^300,307,314,318^
	GPR109A	Niacin	Colorectal cancer	Inhibit glucose transport and glycolysis	^326^
	xCT	Glutamate cysteine	Non-small cell lung cancer	Regulate cancer metastasis	^344^
	AKT	NA	Unknown	Stimulates proliferation and anti-apoptotic responses	^362^
	Cell cycle regulators	8-Hydroxyquinaldic acid	Colon cancer	Inhibit proliferation and mitochondrial activity	^365^
		Cadaverine	Breast cancer	Inhibit cellular movement and invasion	^366,367^

### Epigenetics of protein metabolism

#### Amino acids can directly affect epigenetics through substrates

The amino acids can affect epigenetics by both by itself or through its substrates. Fox example, intracellular L-arginine is a key regulator of central memory T cell metabolic adaptability, survival ability, and anti-tumor activity through metabolomics and proteomics analysis. Elevated L-arginine levels can cause global metabolic changes, including the transition from glycolysis to oxidative phosphorylation in activated T cells, promote central memory-like cells with higher survival production, and increase antitumor activity in a mouse model. Proteomic analysis showed that T cells were metabolically reprogrammed under the influence of elevated intracellular arginine levels. This experiment further explored the sensors that be needed to mediate arginine metabolism and functional response, and found out three proteins: human bromodomain adjacent to zinc finger domain 1B (BAZ1B), PC4 and SFRS1 (splicing factor, arginine/serine-rich1) interacting protein 1 (PSIP1), and translin (tsn).^368^ Human Bromodomain adjacent to zinc finger domain 1B (BAZ1B) is a transcription regulator that contains a prolyl hydroxylase (PHD) domain that binds to methylated histones. PSIP1 is a transcriptional coactivator related to the protection of apoptosis.^369^ Currently, there are studies about anti-tumor therapy targeting arginine, and arginase has been shown to play an anti-tumor effect in pre-clinical and clinical settings.^350,370^

Leucine itself also has an effect on tumorigenesis. The study found that one function of protein leucine residues is to serve as a posttranslational modification site (PTMS), which plays a role in the development of tumors. Studies have determined that the reason why statins can destroy the integrity and invasiveness of tumor cells is partly through the modification of this cysteine (Cys) modification.^371^ Also, a recent study suggested that elevation of L-arginine in mice liver cells are the important characteristics of T2DM/NASH-associated hepatocarcinogenesis.^372^ Another study confirmed that leucine-rich repeat protein 5 (circFBXL5) is highly expressed in breast cancer tissues and cells, and circFBXL5 knockdown inhibited breast cancer by inhibiting cell migration, invasion and promoting apoptosis.^373^ Study also newly indicates that a subset of myeloma identified as an enrichment for basic leucine zipper domain–binding motifs.^374^ Meanwhile, depletion of cystine inhibits pancreatic tumor cell growth through the regulation of ferroptotic death. The down regulation of SLC7A could inhibit the tumor growth and induce ferroptotic cell death, which is validated by the application of cystathionineclyase, and provided a theoretical basis for the clinical practice of the ferroptotic cell death-inducing drug.^375^

Amino acid derivatives are also involved in epigenetics. Branched-chain amino acids (leucine, isoleucine, and valine) and lysine are decomposed into acetyl-CoA, which may be used by histone acetyl-transferases (HATs).^376^ Importantly, the collection of acetyl-CoA from amino acids also regulates protein acetylation and can promote tumor growth. Leucine provides acetyl-CoA to the recombinant E1A binding protein P300 (EP300) acetyltransferase, which mediates inhibitory acetylation of the mTORC1 regulator Raptor at K1097 and leads to mTORC1 activation.^377^ As mentioned before, mTORC1 plays an important role in the development of tumors. At the same time, branched-chain amino acids were found to play an important role in the treatment of cancer. BCAA-degrading enzyme branched-chain-amino-acid aminotransferases1 (BCAT1) has become a useful prognostic cancer marker.^378–380^ In glioblastoma, BCAA (branched-chain amino acids) catabolism is up-regulated, and this up-regulation depends on BCAT (branched-chain-amino-acid aminotransferases)^381,382^ indicates that targeting bcat1 treatment may have a good therapeutic window in bcat1 dependent tumor.^383^ For example, α-ketoglutarate kills dehydrogenase wild-type glioblastoma cells when BCAT1 protein is lost, which is reversed by re-expression of BCAT1 or supplementation with branched-chain α-ketoacids, downstream metabolic products of BCAT1, which means that the cotreatment of BCAT1 inhibitor gabapentin and AKG results in synthetic lethality.^384^ Meanwhile, another new posttranscriptional regulator of BCAT1 expression is identified in chronic myeloid leukemia (CML), the musashi RNA binding protein 2 (MSI2), which coexpressed in CML blast crisis with BCAT1, which means that MSI2–BCAT1 axis is an important mechanism in driving cancer progression in CML.^385^

Lysine-derived acetyl-CoA plays an important role in the self-renewal of colorectal cancer tumor initiating cells. CD110 is a hemagglutinin (TPO) reactive homodimeric receptor and is expressed in colon tumor initiating cells. When binding with TPO, CD110 activates lysine degradation, thereby producing acetyl-CoA, which is used for acetylation of low density lipoprotein receptor-related protein6 (LRP6) under EP300 (E1A Binding Protein P300) dependence. LRP6 is a co-receptor for Wingless/Integrated (WNT) signaling and is essential in the renewal of colon tumor initiating cells. The acetylation of LRP6 stimulates the activity of LRP6 and the self-renewal of CD110 colon tumor initiating cells.^386^

Amino acid derivative polyamines also can affect tumorigenesis. Polyamines (putrescine, spermine, and spermidine) may be the most famous metabolites that promote tumor proliferation and invasiveness. Increased levels of polyamines have been observed in cancer patients. Polyamines and their metabolites in urine and plasma can be used for cancer diagnosis, and can also be used as a marker of lung cancer and liver tumor progression.^386,387^ Meanwhile, inhibiting polyamine metabolism can inhibit tumor growth by stimulating anti-tumor immunity.^388^ Polyamines affect many processes of tumorigenesis, partly through the regulation of the expression of specific genes through transcription. It acts as charged cations at the pH body and can be associated with nucleic acids,^389^ which in turn affects the global chromatin structure and specific DNA-protein interactions, thereby affecting gene transcription.^390,391^ The posttranscriptional aspects of gene regulation mediated by polyamines are related to eukaryotic translation initiation factor 5A (eFI5A), and its expression/function will adversely affect the prognosis of various cancers.^392^ For example, eFI5A is found as one of the differentially expressed proteins in both glioblastoma and neuroblastoma cells.^393^ MS13 is one of the curcumin analogs that has the potent cytotoxicity and anti-proliferative effects on human glioblastoma U-87 MG and neuroblastoma SH-SY5Y cells through affecting eFI5A.^393^

Moreover, it’s also reported that accumulation of cellular acetyl-CoA promoted de novo lipid biosynthesis and histone H3K27 acetylation, which ultimately regulates the peptidyl arginine deiminase 1 (PADI1)-MAPK-MMP-2/9 pathway. This pathway can serve as a potential therapeutic target in nasopharyngeal carcinoma.^394^

#### Amino acids can indirectly regulate enzymes related to epigenetics

The abundance of serine in the body is also related to the occurrence of tumors.^395^ For example, study found that serine upregulated in acute myeloid leukemia,^396^ as well as primary melanomas.^397^ Serine has recently been identified as an allosteric effect factor of M2 isoform of pyruvate kinase (PKM2).^398^ PKM2 is often upregulated in cancer and has many carcinogenic effects.^399^ For example, by proteomic analysis, it’s demonstrated that PKM2 was identified as key molecules regulated by Wnt/β-catenin signaling in colorectal cancer.^400^ Especially in the drug-resistant colorectal cancer, the sponge for the PKM2-targeting miR-122 may be a potential novel therapeutic target.^401^ Cisplatin, tamoxifen, and doxorubicin were found to affect tyrosine and alanine in breast cancer cell lines, which may contribute to drug resistance.^402^

Serine may link with the metastatic progression of tumor. If serine levels are low, the activity of PKM2 will be reduced, causing glucose-derived carbon to shuttle towards the pathway of serine biosynthesis. However, if the serine level is high, PKM2 is fully active, it will promote a higher rate of glycolysis.^387^ This is very important in the development of tumors. PKM2 can be transferred to the nucleus and form a complex with transforming growth factor β signaling inhibitory factor TGFβ-induced factor (TGIF2) and histone deacetylase 3 (HDAC3), and this complex will bind to the cadherin1 (CDH1) promoter and deacetylate it, thereby inhibiting the expression of e-cadherin, so that epithelial cells will lose their mediated cell adhesion and become invasive.^403^ Arginine may also play special roles in certain tumor such as melanoma and hepatocellular carcinoma (HCC) through Arg auxotrophy.^404^ PEGylated Arg deiminase (ADI-PEG20) breaks down Arg into citrulline and eliminates Arg in the tumor microenvironment, which result in the Arg-auxotrophic tumor cell death,^405^ thus ADI-PEG20 serves as a potential target treatment and improves progression-free survival in patients with Arg-auxotrophic mesothelioma.^406^

Amino acids can provide metabolic intermediates for epigenetics and then regulate related enzymes. There are many kinds of metabolic intermediates related to tumors, and the current research focuses on these five kinds: acetyl-CoA, SAM, FAD, NAD ^+^and tetrahydrofolate(THF).^407^ Among them, *S*-adenosylmethionine (SAM) is related to the induction of amino acid metabolites. As a methylated methyl donor, methionine is the main amino acid that promotes epigenetic regulation. Both DNA methyltransferase (DNMTs) and histone methyltransferase (HMTs) use SAM as the methyl donor. SAM is produced by methionine adenosine transferase (MAT) using methionine and ATP as substrates in the methionine cycle,^408^ which is related to an excess methionine, cysteine, and sulfide in body.^409^ After the donor methyl group, SAM becomes s-adenosyl-homocysteine (SAH), meanwhile it inhibits DNMTs and HMTS. Therefore, changes in the SAM/SAH ratio regulate the activity of these methyltransferases.^410^ The importance of SAM in tumor survival has been found in various studies, but it is worth noting that the unique requirement of cancer cells for methionine is based on SAM dependence, not methionine dependence.^411^ The enhanced methionine cycle leads to an excessive supply of SAM, which leads to DNA hypermethylation and inappropriate gene silencing, as well as abnormal histone methylation and promotion of tumor growth.^412^ Threonine dehydrogenase (TDH)-mediated threonine catabolism can also provide precursors for SAM, which means the upregulation of threonine may increase the possibility of tumor development.^413^ Besides, it’s found that methionine cycle flux specifically influences the epigenetic state of cancer cells and drives tumor initiation. methionine cycle enzymes are enriched in many tumor types.^414^ Although serine is not directly involved in DNA/RNA methylation, serine starvation reduces the DNA/RNA methylation level of cancer cells because of the lack of homocysteine methionine regeneration.^412^ Then the subsequent mechanism is described above (Table 2). As in the experiment, the restriction of dietary serine and glycine can reduce tumor growth in xenograft and allograft models.^412^

**Table 2:** Epigenetic regulation mediated metabolites in cancers

Category	Metabolites	Effect target	Effects mediated by target	Ref./refs.
Glucose	Glucose	Histone H3	Tumor formation Tumor proliferation	^41,44^
		NEDD4	Activate cancer-related pathways	^41^
		PKM2	Tumor growth	^45^
		OGT	Tumor proliferation Altering tumor metabolism Metastasis	^55,58–60^
		Histone 2A K119	Glucose starvation-induced cell death	^70,71^
		ncRNA	Promotes cell survival (NBR2) Modulate key glycolytic enzymes in tumors Inhibition of PI3K/Akt/mTOR signaling Tumor suppressor MEG)	^75–83^
Fatty acid	Short-chain fatty acid	HDAC	Tumor suppressor gene expression; MicroRNA expression; Activation, nuclear translocation and DNA binding of transcription factors; Cell cycle arrest and cell apoptosis HDAC transcription inhibition	^118,213,216,218–220^
		KAT	MHC class I related chain A/B expression	^220^
	Medium-chain fatty acids	HDAC	Tumor suppressor gene expression	^234,235^
	Acetone body	HDAC	Inhibition of lipolysis, inflammation, oxidative stress, cancer growth, and angiogenesis	^237^
	ω-3 polyunsaturated fatty acid	EZH2	H3K27me3 modification MicroRNA silencing regulation	^216,253^
		CD44,MMP-9	Extracellular matrix (ECM) remodeling inhibition	^254,255^
		Sirtuin 1	Histones and non-histone proteins Deacetylase	^257,258^
		miRNA-26	Contribution of 15-PGDH’s translation	^265–267^
		miRNAs	Exosome secretion of miRNAs	^269^
	Fish oil and pectin	Genes involved in apoptosis	Cell apoptosis	^259–261^
	DHA and butyric acid	Apoptosis related-genes promoters	Inhibition of apoptosis-related genes Methylation	^263^
	Fatty acid ester of phloridzin	HDAC	Inhibition of HDAC expression	^270^
	DMU-214	Migration and proliferation related factors	Inhibition of cell proliferation and movement	^275^
		GPR56	Inhibition of cell motility	^276^
	Protopanaxadiol	Puma, Noxa,MCL-1	Cancer cell death	^277^
		Fatty acid and cholesterol biosynthesis related factors	Cancer cell death	^277^
	Acetyl-CoA	NA	Histone acetylation	^287,289^
	Organic acid	NA	Histone acylation	^295–299^
Amino acid	L-arginine	BAZ1B	Methylated histones	^369,370^
		Psip1	The protection of apoptosis	^369,370^
		circFBXL5	Inhibiting cell migration, invasion and promoting apoptosis	^375^
	Acetyl-CoA	NA	Histone acetylation	^377^
	Lysine-derived acetyl-CoA	LRP6	Wnt signaling and renewal of colon tumor initiating cells	^387^
	Polyamines	eFI5A	Effect posttranscriptional aspects of gene regulation	^393^
	Serine	PKM2	Key molecules regulated by Wnt/β-catenin signal	^401^
		HDAC3	Histone deacetylation	^404^
		TGIF2	Inhibited transforming growth factor Β signal	^404^
	*S*-adenosylmethionine	DNMTs	DNA methyltransferase	^409^
		HMTs	Histone methyltransferase	^409^

As mentioned earlier, amino acids can affect the development of tumors by regulating epigenetics in cells. Through the study of the mechanism, it can better provide new basis for clinical diagnosis and treatment, thereby promoting the progress of tumor diagnosis and treatment. L-asparaginase is a bacteria-derived enzyme that catalyzes the degradation of asparagine into ammonia and aspartate, which plays a important role in leukemia cells.^415^ As a result, L-asparaginase is an anticancer agent targeting the amino acid metabolism to treat acute lymphoblastic leukemia (ALL)^416^ and extranodal NK/T cell lymphoma.^417^

## Conclusion

Abnormal cell metabolism has been recognized as a sign of cancer biology. The hallmark of cancer metabolism change is the re-engineering of energy-generating pathways and increased production of biosynthetic intermediates to meet the rapidly proliferating needs of the tumor cells. The dynamic changes of intracellular and extracellular metabolites, especially changes in the concentration of nutrients, can regulate cell signal transduction and gene expression at the epigenetic level through the metabolite sensing mechanism, creating a favorable microenvironment for cancer cells. In this review, we systematically summarize metabolite-sensing mechanism in cancer cells and the signal transduction and epigenetics affected by it and summarize the therapeutic targets for cancers which is related to metabolite sensing as well as the recent advances of target drugs (Table 3). It not only further perfects the metabolism and growth mechanism of cancer cells, but also provides a feasible orientation for the research of tumor-targeted drugs, creating a new direction for cancer prevention and treatment.

**Table 3:** Target therapy related with metabolite sensing in cancers

Category	Effect target	Targeted medicine	Targeted effect	Ref./refs.
Glucose	GLUT	Phloretin	Inhibitor	^86^
		Fasentin		^86^
		STF-31		^86^
		WZB117		^86^
		Ritonavir		^86^
		Silybin		^87^
	HK	2-DG	Inhibitor	^88,89^
		LN	Inhibitor	^90^
		Resveratrol	Inhibitor	^91^
		Astragalin	Antagonist	^92^
		Chrysin	Antagonist	^93^
		Genistein-27	Antagonist	^94^
		Benserazide	Inhibitor	^95^
	PI3K/Akt	Afuresertib	Inhibitor	^96^
		Uprosertib		
		Ipatasertib		
	mTORC1/2	PP242	Inhibitor	^101^
	PI3K/mTOR	NVP-BEZ235	Inhibitor	^102^
Fatty acid	GPR109A	MK1903	Antagonist	^141^
		Acifran		^142^
		5-ANA		^143^
		GSK256073		^144^
	Fatty acid β oxidation	Etomuslim	Antagonist	^136^
		Peroxicillin		
	LDHA	FX11	Inhibitor	^150,152^
		*N*-hydroxy-2-carboxy-substituted indole compounds		^150,153^
	GPR43	4-CMTB	Agonist	^167–170^
		AZ1729		^167–170^
	GPR84	Compound 33	Antagonist	^174^
		GLPG1205		^175^
		LY-237	Agonist	^178^
		6-Octylaminouracil		^179^
	GPR40	GW100	Antagonist	^184^
		DC260126		^185^
	GPR120	AH7614	Antagonist	^187,188^
		GW9508	Agonist	^190^
		TUG-891		^192^
		Compound 39		^193^
	GPR78	Anti-CTD GPR78 antibody	Blocker	^197^
		miR-936	Antagonist	^198^
	HDAC	Vorinostat	Inhibitor	^223–225^
		Entinostat		^226^
		Valproate		^227^
		Romidepsin		^228^
	CD44	CD44 mono-antibody	Blocker	^256^
	15-PGDH	HDAC inhibitors	Agonist	^269^
		Nonsteroidal anti-inflammatory drugs		
		PPAR-γ agonists		
	FASN	Cerulenin	Inhibitor	^278^
	ACSS2	MTB-9655	Inhibitor	NCT04990739
	ACC1	TOFA	Inhibitor	^285^
		ND-646		^286,287^
Amino acid	mTOR	Temsirolimus	Inhibitor	^322^
		eEF-2K		^323^
	GPR109A	Niacin	Agonist	^324^
	GPR56	Transglutaminase 2	Inhibitor	^332^
		LGR5	Agonist	^340^
	Glutamine	ASCT2	Agonist	^355^
		v-9302	Inhibitor	^356^
	Leucine	circFBXL5	Agonist	^374^
	mTORC1	EP300	Agonist	^378^
	BCAT	BCAA	Agonist	^382,383^
	Serine	PKM2	Agonist	^388^

## Future perspectives

In the past few decades, research on the metabolite sensing mechanism has received great attention. In recent years, the metabolite sensing mechanism in cancer has become one of the hot topics, but there are still many problems to be solved. The most thoroughly studied at present is the metabolite-sensitive GPCRs. Their ligands involve three major nutrients as well as their intermediate metabolites. Based on specific functions, metabolite-sensitive GPCRs provide an interesting connection between nutrition, metabolism, gastroenterology, microbiology, and immunology. These receptors provide great potential for understanding how cancer development is closely linked to metabolism. However, previous studies have focused on its role in intestinal immunity and inflammation, while less research has been done in cancers. Is its role in immunity and inflammation related to tumor origination and development? Except in the intestine, do GPCRs play the same role in tumors in other locations? Does metabolite-sensitive GPCR mediate homing and chemotaxis of leukocytes within? Can we develop metabolite-sensing GPCR tool compounds with drug-like properties? Can we adjust the diet to reduce cancer? How can we achieve these? All of the above still need further study. In addition, there is still a large gap for targeted drugs targeting metabolite sensor GPCRs, and their application for cancer therapy is limited due to their bilateral physiological and pathological effects. If these targeted drugs can be spatially targeted to tumors’ space, this will have a huge impact on cancer therapy. At the same time, our review divides cell metabolism into three parts: glucose, fatty acid, and amino acid, in fact, these nutrients share a common metabolic pathway partly. It will be meaningful to find potential therapeutic targets on the common metabolic pathway. In addition, for the current research on fatty acid conjugated sensing mechanism, lipid attachment of proteins involves a variety of lipid types, such as myristate, palmitate, and cholesterol, but the current research focuses on histone modification sites, what is the specific role for histone PTM in the development of tumors? Other than the three major nutrients and their intermediate metabolites, other substances that can affect signal transduction or epigenetics through metabolite sensing mechanisms, such as polyphenols and glucosinolates in the diet. Epidemiology studies have shown that they can effectively reduce the risk of various cancers including prostate cancer and breast cancer, so what is the specific mechanism? Except for these metabolites, are there other substances that can play a role in cancer through metabolite sensing mechanisms? There are a variety of immune cells in the tumor microenvironment, such as macrophages, T cells, etc. How metabolites affect these immune cells and how oncometabolites contribute to tumor immune evasion by affecting the function of immune cells still need to be further summarized. Also, whether the abnormality of metabolite sensors on immune cells affect their function is still unclear. Finally, the kidney has an effective sensing and signal transmission mechanism. The organic anion transporter (OAT) of the 22 family members of the kidney solute carrier can transport various waste solutes and plays an important role in the kidney metabolite sensing mechanism. However, current researches are mostly concentrated on OAT1, so what is the contribution of OAT3 in metabolite sensing and regulation of renal excretion pathways? This may beyond our imagination.

## Data Availability

Please contact the corresponding author for all data requests.

## References

[1] Vander Heiden MG, Cantley LC, Thompson CB (2009). Understanding the Warburg effect: the metabolic requirements of cell proliferation. Science.

[2] Carracedo A, Cantley LC, Pandolfi PP (2013). Cancer metabolism: fatty acid oxidation in the limelight. Nat. Rev. Cancer.

[3] Zhao Y, Butler EB, Tan M (2013). Targeting cellular metabolism to improve cancer therapeutics. Cell Death Dis..

[4] Vander Heiden MG (2011). Targeting cancer metabolism: a therapeutic window opens. Nat. Rev. Drug Discov..

[5] Menendez JA (2010). Fine-tuning the lipogenic/lipolytic balance to optimize the metabolic requirements of cancer cell growth: molecular mechanisms and therapeutic perspectives. Biochim. Biophys. Acta.

[6] Hino S, Kohrogi K, Nakao M (2016). Histone demethylase LSD1 controls the phenotypic plasticity of cancer cells. Cancer Sci..

[7] Wang YP, Lei QY (2018). Metabolite sensing and signaling in cell metabolism. Signal Transduct. Target. Ther..

[8] Nakai H (2015). Navigating metabolism by Navdeep S. Chandel. Q. Rev. Biol..

[9] Mycielska ME (2006). Citrate enhances in vitro metastatic behaviours of PC-3M human prostate cancer cells: status of endogenous citrate and dependence on aconitase and fatty acid synthase. Int. J. Biochem. Cell Biol..

[10] Xiong Q (2018). Metabolite-sensing G protein coupled receptor TGR5 protects host from viral infection through amplifying type I interferon responses. Front. Immunol..

[11] Yang R (2019). LSH interacts with and stabilizes GINS4 transcript that promotes tumourigenesis in non-small cell lung cancer. J. Exp. Clin. Cancer Res..

[12] Looger LL, Dwyer MA, Smith JJ, Hellinga HW (2003). Computational design of receptor and sensor proteins with novel functions. Nature.

[13] Chantranupong L (2016). The CASTOR proteins are arginine sensors for the mTORC1 pathway. Cell.

[14] Saxton RA (2016). Structural basis for leucine sensing by the Sestrin2-mTORC1 pathway. Science.

[15] Miranda Furtado CL (2019). Epidrugs: targeting epigenetic marks in cancer treatment. Epigenetics.

[16] Katada S, Imhof A, Sassone-Corsi P (2012). Connecting threads: epigenetics and metabolism. Cell.

[17] Werner HM, Mills GB, Ram PT (2014). Cancer systems biology: a peek into the future of patient care?. Nat. Rev. Clin. Oncol..

[18] Teperino R, Lempradl A, Pospisilik JA (2013). Bridging epigenomics and complex disease: the basics. Cell Mol. Life Sci..

[19] Ouyang C (2019). Chromatin remodeling factor lymphoid-specific helicase links with Epstein-Barr virus associated the follicular germinal center B cell lymphomas. J. Cancer Res. Ther..

[20] Zhang L (2019). The interplay of circulating tumor DNA and chromatin modification, therapeutic resistance, and metastasis. Mol. Cancer.

[21] Sun L, Zhang H, Gao P (2022). Metabolic reprogramming and epigenetic modifications on the path to cancer. Protein Cell.

[22] Williams D, Fingleton B (2019). Non-canonical roles for metabolic enzymes and intermediates in malignant progression and metastasis. Clin. Exp. Metastasis.

[23] Wolffe AP, Matzke MA (1999). Epigenetics: regulation through repression. Science.

[24] Marmorstein R, Zhou MM (2014). Writers and readers of histone acetylation: structure, mechanism, and inhibition. Cold Spring Harb. Perspect. Biol..

[25] Wilhelm JA, McCarty KS (1970). The uptake and turnover of acetate in HeLa cell histone fractions. Cancer Res..

[26] Li ST (2020). Myc-mediated SDHA acetylation triggers epigenetic regulation of gene expression and tumorigenesis. Nat. Metab..

[27] Cairns RA, Harris IS, Mak TW (2011). Regulation of cancer cell metabolism. Nat. Rev. Cancer.

[28] Park JW, Han JW (2019). Targeting epigenetics for cancer therapy. Arch. Pharmacal Res..

[29] Nebbioso A, Tambaro FP, Dell’Aversana C, Altucci L (2018). Cancer epigenetics: moving forward. PLoS Genet..

[30] Rask-Andersen M, Almén MSL, Schiöth HB (2011). Trends in the exploitation of novel drug targets. Nat. Rev. Drug Discov..

[31] Lefkowitz RJ (2007). Seven transmembrane receptors: something old, something new. Acta Physiol..

[32] Oldham WM, Hamm HE (2007). How do receptors activate G proteins?. Adv. Protein Chem..

[33] Wettschureck N, Offermanns S (2005). Mammalian G proteins and their cell type specific functions. Physiol. Rev..

[34] Honn KV (2016). 12-HETER1/GPR31, a high-affinity 12(S)-hydroxyeicosatetraenoic acid receptor, is significantly up-regulated in prostate cancer and plays a critical role in prostate cancer progression. FASEB J..

[35] Morita N (2019). GPR31-dependent dendrite protrusion of intestinal CX3CR1(+) cells by bacterial metabolites. Nature.

[36] Kaelin WG (2008). The von Hippel-Lindau tumour suppressor protein: O_2_ sensing and cancer. Nat. Rev. Cancer.

[37] Yang MH (2008). Direct regulation of TWIST by HIF-1alpha promotes metastasis. Nat. Cell Biol..

[38] Mak P (2010). ERbeta impedes prostate cancer EMT by destabilizing HIF-1alpha and inhibiting VEGF-mediated snail nuclear localization: implications for Gleason grading. Cancer Cell.

[39] Mu X (2017). Oncometabolite succinate promotes angiogenesis by upregulating VEGF expression through GPR91-mediated STAT3 and ERK activation. Oncotarget.

[40] Nastasi C (2021). Inhibition of succinate dehydrogenase activity impairs human T cell activation and function. Sci. Rep..

[41] Zhang X (2017). H3 ubiquitination by NEDD4 regulates H3 acetylation and tumorigenesis. Nat. Commun..

[42] Persaud A (2014). Tyrosine phosphorylation of NEDD4 activates its ubiquitin ligase activity. Sci. Signal..

[43] Yoder SM, Dineen SL, Wang Z, Thurmond DC (2014). YES, a Src family kinase, is a proximal glucose-specific activator of cell division cycle control protein 42 (Cdc42) in pancreatic islet beta cells. J. Biol. Chem..

[44] Gupta C, Kaur J, Tikoo K (2014). Regulation of MDA-MB-231 cell proliferation by GSK-3beta involves epigenetic modifications under high glucose conditions. Exp. Cell Res..

[45] Christofk HR (2008). The M2 splice isoform of pyruvate kinase is important for cancer metabolism and tumour growth. Nature.

[46] Roll JD, Rivenbark AG, Jones WD, Coleman WB (2008). DNMT3b overexpression contributes to a hypermethylator phenotype in human breast cancer cell lines. Mol. Cancer.

[47] Dougherty CJ (2008). Selective apoptosis of breast cancer cells by siRNA targeting of BORIS. Biochem. Biophys. Res. Commun..

[48] Clark RJ (2003). Diabetes and the accompanying hyperglycemia impairs cardiomyocyte calcium cycling through increased nuclear O-GlcNAcylation. J. Biol. Chem..

[49] Haltiwanger RS, Grove K, Philipsberg GA (1998). Modulation of O-linked N-acetylglucosamine levels on nuclear and cytoplasmic proteins in vivo using the peptide O-GlcNAc-beta-N-acetylglucosaminidase inhibitor O-(2-acetamido-2-deoxy-D-glucopyranosylidene)amino-N-phenylcarbamate. J. Biol. Chem..

[50] Hart GW (1984). Topography and polypeptide distribution of terminal N-acetylglucosamine residues on the surfaces of intact lymphocytes. Evidence for O-linked GlcNAc. J. Biol. Chem..

[51] Hart GW, Housley MP, Slawson C (2007). Cycling of O-linked beta-N-acetylglucosamine on nucleocytoplasmic proteins. Nature.

[52] Roos MD, Su K, Baker JR, Kudlow JE (1997). O glycosylation of an Sp1-derived peptide blocks known Sp1 protein interactions. Mol. Cell Biol..

[53] Zeidan Q, Wang Z, De Maio A, Hart GW (2010). O-GlcNAc cycling enzymes associate with the translational machinery and modify core ribosomal proteins. Mol. Biol. Cell.

[54] Housley MP (2008). O-GlcNAc regulates FoxO activation in response to glucose. J. Biol. Chem..

[55] Caldwell SA (2010). Nutrient sensor O-GlcNAc transferase regulates breast cancer tumorigenesis through targeting of the oncogenic transcription factor FoxM1. Oncogene.

[56] Lynch TP (2012). Critical role of O-linked β-N-acetylglucosamine transferase in prostate cancer invasion, angiogenesis, and metastasis. J. Biol. Chem..

[57] Ma Z, Vocadlo DJ, Vosseller K (2013). Hyper-O-GlcNAcylation is anti-apoptotic and maintains constitutive NF-κB activity in pancreatic cancer cells. J. Biol. Chem..

[58] Beishline K, Azizkhan-Clifford J (2015). Sp1 and the ‘hallmarks of cancer’. FEBS J..

[59] Duan W (2014). Hyperglycemia, a neglected factor during cancer progression. Biomed. Res. Int.

[60] Vasconcelos-Dos-Santos A, de Queiroz RM, da Costa Rodrigues B, Todeschini AR, Dias WB (2018). Hyperglycemia and aberrant O-GlcNAcylation: contributions to tumor progression. J. Bioenerg. Biomembr..

[61] Chocarro-Calvo A, García-Martínez JM, Ardila-González S, De la Vieja A, García-Jiménez C (2013). Glucose-induced β-catenin acetylation enhances Wnt signaling in cancer. Mol. Cell.

[62] Zhang X (2017). The essential role of YAP O-GlcNAcylation in high-glucose-stimulated liver tumorigenesis. Nat. Commun..

[63] Cao R, Tsukada Y, Zhang Y (2005). Role of Bmi-1 and Ring1A in H2A ubiquitylation and Hox gene silencing. Mol. Cell.

[64] Scheuermann JC, Gutierrez L, Muller J (2012). Histone H2A monoubiquitination and Polycomb repression: the missing pieces of the puzzle. Fly.

[65] Wang H (2004). Role of histone H2A ubiquitination in Polycomb silencing. Nature.

[66] Gil J, O’Loghlen A (2014). PRC1 complex diversity: where is it taking us?. Trends Cell Biol..

[67] Blackledge NP, Rose NR, Klose RJ (2015). Targeting Polycomb systems to regulate gene expression: modifications to a complex story. Nat. Rev. Mol. Cell Biol..

[68] Gray F (2016). BMI1 regulates PRC1 architecture and activity through homo- and hetero-oligomerization. Nat. Commun..

[69] Yong KJ (2016). Targeted BMI1 inhibition impairs tumor growth in lung adenocarcinomas with low CEBPalpha expression. Sci. Transl. Med..

[70] Ganaie AA (2018). BMI1 drives metastasis of prostate cancer in Caucasian and African-American men and is a potential therapeutic target: hypothesis tested in race-specific models. Clin. Cancer Res..

[71] Bansal N (2016). BMI-1 targeting interferes with patient-derived tumor-initiating cell survival and tumor growth in prostate cancer. Clin. Cancer Res..

[72] Senft D, Ronai ZA (2015). UPR, autophagy, and mitochondria crosstalk underlies the ER stress response. Trends Biochem. Sci..

[73] Zhang Y (2020). H2A monoubiquitination links glucose availability to epigenetic regulation of the endoplasmic reticulum stress response and cancer cell death. Cancer Res..

[74] Farooqi AA, Tabassum S, Ahmad A (2017). MicroRNA-34a: a versatile regulator of myriads of targets in different cancers. Int. J. Mol. Sci..

[75] Joost HG (2002). Nomenclature of the GLUT/SLC2A family of sugar/polyol transport facilitators. Am. J. Physiol. Endocrinol. Metab..

[76] Mueckler M, Thorens B (2013). The SLC2 (GLUT) family of membrane transporters. Mol. Asp. Med..

[77] Liu X, Gan B (2016). lncRNA NBR2 modulates cancer cell sensitivity to phenformin through GLUT1. Cell Cycle.

[78] Zhang J (2013). Loss of microRNA-143/145 disturbs cellular growth and apoptosis of human epithelial cancers by impairing the MDM2-p53 feedback loop. Oncogene.

[79] Volinia S (2006). A microRNA expression signature of human solid tumors defines cancer gene targets. Proc. Natl Acad. Sci. USA.

[80] Kano M (2010). miR-145, miR-133a and miR-133b: tumor-suppressive miRNAs target FSCN1 in esophageal squamous cell carcinoma. Int. J. Cancer.

[81] Kawakami K (2012). The functional significance of miR-1 and miR-133a in renal cell carcinoma. Eur. J. Cancer.

[82] Kefas B (2010). Pyruvate kinase M2 is a target of the tumor-suppressive microRNA-326 and regulates the survival of glioma cells. Neuro Oncol..

[83] Guo H (2016). miRNA-451 inhibits glioma cell proliferation and invasion by downregulating glucose transporter 1. Tumour Biol..

[84] Shankaraiah RC, Veronese A, Sabbioni S, Negrini M (2018). Non-coding RNAs in the reprogramming of glucose metabolism in cancer. Cancer Lett..

[85] Chen L (2019). DNA methylation modifier LSH inhibits p53 ubiquitination and transactivates p53 to promote lipid metabolism. Epigenetics Chromatin.

[86] Abdel-Wahab AF, Mahmoud W, Al-Harizy RM (2019). Targeting glucose metabolism to suppress cancer progression: prospective of anti-glycolytic cancer therapy. Pharmacol. Res..

[87] Flaig TW (2007). A phase I and pharmacokinetic study of silybin-phytosome in prostate cancer patients. Invest. N. Drugs.

[88] Aghaee F, Pirayesh Islamian J, Baradaran B (2012). Enhanced radiosensitivity and chemosensitivity of breast cancer cells by 2-deoxy-d-glucose in combination therapy. J. Breast Cancer.

[89] Zhang D (2014). 2-Deoxy-D-glucose targeting of glucose metabolism in cancer cells as a potential therapy. Cancer Lett..

[90] Di Cosimo S (2003). Lonidamine: efficacy and safety in clinical trials for the treatment of solid tumors. Drugs Today.

[91] Dai W (2015). By reducing hexokinase 2, resveratrol induces apoptosis in HCC cells addicted to aerobic glycolysis and inhibits tumor growth in mice. Oncotarget.

[92] Li W (2017). Astragalin reduces hexokinase 2 through increasing miR-125b to inhibit the proliferation of hepatocellular carcinoma cells in vitro and in vivo. J. Agric. Food Chem..

[93] Xu D (2017). Chrysin inhibited tumor glycolysis and induced apoptosis in hepatocellular carcinoma by targeting hexokinase-2. J. Exp. Clin. Cancer Res..

[94] Tao L (2017). Gen-27, a newly synthesized flavonoid, inhibits glycolysis and induces cell apoptosis via suppression of hexokinase II in human breast cancer cells. Biochem. Pharm..

[95] Li W (2017). Benserazide, a dopadecarboxylase inhibitor, suppresses tumor growth by targeting hexokinase 2. J. Exp. Clin. Cancer Res..

[96] Nitulescu GM (2016). Akt inhibitors in cancer treatment: the long journey from drug discovery to clinical use (Review). Int. J. Oncol..

[97] Dienstmann R, Rodon J, Serra V, Tabernero J (2014). Picking the point of inhibition: a comparative review of PI3K/AKT/mTOR pathway inhibitors. Mol. Cancer Ther..

[98] Chan J, Kulke M (2014). Targeting the mTOR signaling pathway in neuroendocrine tumors. Curr. Treat. Options Oncol..

[99] de Melo AC, Paulino E, Garces ÁH (2017). A review of mTOR pathway inhibitors in gynecologic cancer. Oxid. Med. Cell Longev..

[100] Mendoza MC, Er EE, Blenis J (2011). The Ras-ERK and PI3K-mTOR pathways: cross-talk and compensation. Trends Biochem. Sci..

[101] Zhang YJ, Duan Y, Zheng XF (2011). Targeting the mTOR kinase domain: the second generation of mTOR inhibitors. Drug Discov. Today.

[102] Hsu CM (2018). NVP-BEZ235, a dual PI3K-mTOR inhibitor, suppresses the growth of FaDu hypopharyngeal squamous cell carcinoma and has a synergistic effect with Cisplatin. Cell Death Discov..

[103] Maher M, Diesch J, Casquero R, Buschbeck M (2018). Epigenetic-transcriptional regulation of fatty acid metabolism and its alterations in leukaemia. Front. Genet..

[104] Wang PY (2021). Reducing fatty acid oxidation improves cancer-free survival in a mouse model of Li-Fraumeni syndrome. Cancer Prev. Res..

[105] Nallanthighal S (2020). Inhibition of collagen XI alpha 1-induced fatty acid oxidation triggers apoptotic cell death in cisplatin-resistant ovarian cancer. Cell Death Dis..

[106] Stine ZE, Schug ZT, Salvino JM, Dang CV (2022). Targeting cancer metabolism in the era of precision oncology. Nat. Rev. Drug Discov..

[107] Tan B (2020). Identifying potential serum biomarkers of breast cancer through targeted free fatty acid profiles screening based on a GC-MS platform. Biomed. Chromatogr..

[108] Zheng Q (2021). Neutral desorption extractive electrospray ionization mass spectrometry analysis sputum for non-invasive lung adenocarcinoma detection. Onco Targets Ther..

[109] Wilcox M (2021). A 16-Channel (13)C array coil for magnetic resonance spectroscopy of the breast at 7T. IEEE Trans. Biomed. Eng..

[110] Le Poul E (2003). Functional characterization of human receptors for short chain fatty acids and their role in polymorphonuclear cell activation. J. Biol. Chem..

[111] Taggart AK (2005). (D)-beta-Hydroxybutyrate inhibits adipocyte lipolysis via the nicotinic acid receptor PUMA-G. J. Biol. Chem..

[112] Thangaraju M (2009). GPR109A is a G-protein-coupled receptor for the bacterial fermentation product butyrate and functions as a tumor suppressor in colon. Cancer Res..

[113] Wang J, Wu X, Simonavicius N, Tian H, Ling L (2006). Medium-chain fatty acids as ligands for orphan G protein-coupled receptor GPR84. J. Biol. Chem..

[114] Smith PM (2013). The microbial metabolites, short-chain fatty acids, regulate colonic Treg cell homeostasis. Science.

[115] Singh N (2014). Activation of Gpr109a, receptor for niacin and the commensal metabolite butyrate, suppresses colonic inflammation and carcinogenesis. Immunity.

[116] Brestoff JR, Artis D (2013). Commensal bacteria at the interface of host metabolism and the immune system. Nat. Immunol..

[117] Melhem H, Kaya B, Ayata CK, Hruz P, Niess JH (2019). Metabolite-sensing G protein-coupled receptors connect the diet-microbiota-metabolites axis to inflammatory bowel disease. Cells.

[118] Thorburn AN, Macia L, Mackay CR (2014). Diet, metabolites, and “western-lifestyle” inflammatory diseases. Immunity.

[119] Richards JL, Yap YA, McLeod KH, Mackay CR, Marino E (2016). Dietary metabolites and the gut microbiota: an alternative approach to control inflammatory and autoimmune diseases. Clin. Transl. Immunol..

[120] Oh DY (2010). GPR120 is an omega-3 fatty acid receptor mediating potent anti-inflammatory and insulin-sensitizing effects. Cell.

[121] Xie, J., Wang, X. & Proud, C. G. mTOR inhibitors in cancer therapy. *F1000Res* **5**, F1000 Faculty Rev-2078 (2016). 10.12688/f1000research.9207.1 PMC5007757 27635236

[122] Rasoamanana R, Darcel N, Fromentin G, Tome D (2012). Nutrient sensing and signalling by the gut. Proc. Nutr. Soc..

[123] Plotnikov A (2015). The nuclear translocation of ERK1/2 as an anticancer target. Nat. Commun..

[124] Chapnick DA, Warner L, Bernet J, Rao T, Liu X (2011). Partners in crime: the TGFbeta and MAPK pathways in cancer progression. Cell Biosci..

[125] Laplante M, Sabatini DM (2009). An emerging role of mTOR in lipid biosynthesis. Curr. Biol..

[126] Peterson TR (2011). mTOR complex 1 regulates lipin 1 localization to control the SREBP pathway. Cell.

[127] Kim JE, Chen J (2004). regulation of peroxisome proliferator-activated receptor-gamma activity by mammalian target of rapamycin and amino acids in adipogenesis. Diabetes.

[128] Zhang HH (2009). Insulin stimulates adipogenesis through the Akt-TSC2-mTORC1 pathway. PLoS ONE.

[129] Lefkowitz RJ, Shenoy SK (2005). Transduction of receptor signals by beta-arrestins. Science.

[130] Walters RW (2009). beta-Arrestin1 mediates nicotinic acid-induced flushing, but not its antilipolytic effect, in mice. J. Clin. Investig..

[131] Lee SU (2013). beta-Arrestin 2 mediates G protein-coupled receptor 43 signals to nuclear factor-kappaB. Biol. Pharm. Bull..

[132] Li Y, Kokrashvili Z, Mosinger B, Margolskee RF (2013). Gustducin couples fatty acid receptors to GLP-1 release in colon. Am. J. Physiol. Endocrinol. Metab..

[133] Kimura I, Ichimura A, Ohue-Kitano R, Igarashi M (2020). Free fatty acid receptors in health and disease. Physiol. Rev..

[134] Gao H (2004). Identification of beta-arrestin2 as a G protein-coupled receptor-stimulated regulator of NF-kappa B pathways. Mol. Cell.

[135] Gaidarov I (2013). Differential tissue and ligand-dependent signaling of GPR109A receptor: implications for anti-atherosclerotic therapeutic potential. Cell. Signal..

[136] Staubert C, Broom OJ, Nordstrom A (2015). Hydroxycarboxylic acid receptors are essential for breast cancer cells to control their lipid/fatty acid metabolism. Oncotarget.

[137] Ristic B, Bhutia YD, Ganapathy V (2017). Cell-surface G-protein-coupled receptors for tumor-associated metabolites: a direct link to mitochondrial dysfunction in cancer. Biochim. Biophys. Acta Rev. Cancer.

[138] Elangovan S (2014). The niacin/butyrate receptor GPR109A suppresses mammary tumorigenesis by inhibiting cell survival. Cancer Res..

[139] Santolla MF (2014). Niacin activates the G protein estrogen receptor (GPER)-mediated signalling. Cell. Signal..

[140] Sivaprakasam S, Prasad PD, Singh N (2016). Benefits of short-chain fatty acids and their receptors in inflammation and carcinogenesis. Pharmacol. Ther..

[141] Viatchenko-Karpinski V, Kong L, Weng HR (2022). Activation of microglial GPR109A alleviates thermal hyperalgesia in female lupus mice by suppressing IL-18 and glutamatergic synaptic activity. Glia.

[142] Adepu KK, Kachhap S, Bhandari D, Anishkin A, Chintapalli SV (2022). Computational insights on molecular interactions of acifran with GPR109A and GPR109B. J. Mol. Model.

[143] Jeong S (2020). 5-Aminosalicylic acid Azo-coupled with a GPR109A agonist is a colon-targeted anticolitic codrug with a reduced risk of skin toxicity. Mol. Pharm..

[144] Olson EJ (2019). A randomized, placebo-controlled trial to assess the effects of 8 weeks of administration of GSK256073, a selective GPR109A agonist, on high-density lipoprotein cholesterol in subjects with dyslipidemia. Clin. Pharm. Drug Dev..

[145] Offermanns S (2014). Free fatty acid (FFA) and hydroxy carboxylic acid (HCA) receptors. Annu. Rev. Pharm. Toxicol..

[146] Offermanns S (2011). International Union of Basic and Clinical Pharmacology. LXXXII: Nomenclature and classification of hydroxy-carboxylic acid receptors (GPR81, GPR109A, and GPR109B). Pharm. Rev..

[147] Roland CL (2014). Cell surface lactate receptor GPR81 is crucial for cancer cell survival. Cancer Res..

[148] Ahmed K (2010). An autocrine lactate loop mediates insulin-dependent inhibition of lipolysis through GPR81. Cell Metab..

[149] Brown TP, Ganapathy V (2020). Lactate/GPR81 signaling and proton motive force in cancer: role in angiogenesis, immune escape, nutrition, and Warburg phenomenon. Pharmacol. Ther..

[150] Miao P, Sheng S, Sun X, Liu J, Huang G (2013). Lactate dehydrogenase A in cancer: a promising target for diagnosis and therapy. IUBMB Life.

[151] Sheng H, Tang W (2016). Glycolysis inhibitors for anticancer therapy: a review of recent patents. Recent Pat. Anticancer Drug Discov..

[152] Le A (2010). Inhibition of lactate dehydrogenase A induces oxidative stress and inhibits tumor progression. Proc. Natl Acad. Sci. USA.

[153] Porporato PE, Dhup S, Dadhich RK, Copetti T, Sonveaux P (2011). Anticancer targets in the glycolytic metabolism of tumors: a comprehensive review. Front. Pharm..

[154] Lee YJ (2016). G-protein-coupled receptor 81 promotes a malignant phenotype in breast cancer through angiogenic factor secretion. Oncotarget.

[155] Le Floch R (2011). CD147 subunit of lactate/H + symporters MCT1 and hypoxia-inducible MCT4 is critical for energetics and growth of glycolytic tumors. Proc. Natl Acad. Sci. USA.

[156] Martinez-Outschoorn UE (2012). Ketone body utilization drives tumor growth and metastasis. Cell Cycle.

[157] Ahmed K (2009). Deorphanization of GPR109B as a receptor for the beta-oxidation intermediate 3-OH-octanoic acid and its role in the regulation of lipolysis. J. Biol. Chem..

[158] Tang Y, Chen Y, Jiang H, Robbins GT, Nie D (2011). G-protein-coupled receptor for short-chain fatty acids suppresses colon cancer. Int. J. Cancer.

[159] Pan P (2018). Loss of FFAR2 promotes colon cancer by epigenetic dysregulation of inflammation suppressors. Int. J. Cancer.

[160] Kamp ME (2016). G protein-coupled receptor 43 modulates neutrophil recruitment during acute inflammation. PLoS ONE.

[161] Sina, C. et al. S1640 G-protein coupled receptor 43 (Gpr43) is essential for neutrophil recruitment during intestinal inflammation. *J. Immunol*. **183**, 7514–7522 (2009). 10.4049/jimmunol.0900063 19917676

[162] Vinolo MAR (2009). Short-chain fatty acids stimulate the migration of neutrophils to inflammatory sites. Clin. Sci..

[163] Lavoie S (2020). Expression of free fatty acid receptor 2 by dendritic cells prevents their expression of interleukin 27 and is required for maintenance of mucosal barrier and immune response against colorectal tumors in mice. Gastroenterology.

[164] Fachi JL (2020). Acetate coordinates neutrophil and ILC3 responses against *C. difficile* through FFAR2. J. Exp. Med..

[165] Chun E (2019). Metabolite-sensing receptor Ffar2 regulates colonic group 3 innate lymphoid cells and gut immunity. Immunity.

[166] Cosin-Roger J, Ortiz-Masia D, Barrachina MD, Calatayud S (2020). Metabolite sensing GPCRs: promising therapeutic targets for cancer treatment?. Cells.

[167] Zhang W, Wang W, Xu M, Xie H, Pu Z (2021). GPR43 regulation of mitochondrial damage to alleviate inflammatory reaction in sepsis. Aging.

[168] Dewulf EM (2013). Evaluation of the relationship between GPR43 and adiposity in human. Nutr. Metab..

[169] Smith NJ (2011). Extracellular loop 2 of the free fatty acid receptor 2 mediates allosterism of a phenylacetamide ago-allosteric modulator. Mol. Pharm..

[170] Bolognini D (2016). A novel allosteric activator of free fatty acid 2 receptor displays unique Gi-functional bias. J. Biol. Chem..

[171] Dietrich PA (2014). GPR84 sustains aberrant beta-catenin signaling in leukemic stem cells for maintenance of MLL leukemogenesis. Blood.

[172] Chen T, Zhang J, Wang Y, Zhou H (2022). Identification of survival-related genes in acute myeloid leukemia (AML) based on cytogenetically normal AML samples using weighted gene coexpression network analysis. Dis. Markers.

[173] Sato N, Meijer L, Skaltsounis L, Greengard P, Brivanlou AH (2004). Maintenance of pluripotency in human and mouse embryonic stem cells through activation of Wnt signaling by a pharmacological GSK-3-specific inhibitor. Nat. Med..

[174] Chen LH (2022). Phosphodiesters as GPR84 antagonists for the treatment of ulcerative colitis. J. Med. Chem..

[175] Labeguere F (2020). Discovery of 9-cyclopropylethynyl-2-((S)-1-[1,4]dioxan-2-ylmethoxy)-6,7-dihydropyrimido[6,1-a]isoquinolin-4-one (GLPG1205), a unique GPR84 negative allosteric modulator undergoing evaluation in a phase II clinical trial. J. Med. Chem..

[176] Recio C (2018). Activation of the immune-metabolic receptor GPR84 enhances inflammation and phagocytosis in macrophages. Front. Immunol..

[177] Kamber RA (2021). Inter-cellular CRISPR screens reveal regulators of cancer cell phagocytosis. Nature.

[178] Pillaiyar T (2018). 6-(Ar)Alkylamino-substituted uracil derivatives: lipid mimetics with potent activity at the orphan G protein-coupled receptor 84 (GPR84). ACS Omega.

[179] Liu Y (2016). Design and synthesis of 2-alkylpyrimidine-4,6-diol and 6-alkylpyridine-2,4-diol as potent GPR84 agonists. ACS Med. Chem. Lett..

[180] Hardy S, St-Onge GG, Joly E, Langelier Y, Prentki M (2005). Oleate promotes the proliferation of breast cancer cells via the G protein-coupled receptor GPR40. J. Biol. Chem..

[181] Liu HH (2022). An SCD1-dependent mechanoresponsive pathway promotes HCC invasion and metastasis through lipid metabolic reprogramming. Mol. Ther..

[182] Usman S, Khawer M, Rafique S, Naz Z, Saleem K (2020). The current status of anti-GPCR drugs against different cancers. J. Pharm. Anal..

[183] Liotti A (2018). Oleic acid promotes prostate cancer malignant phenotype via the G protein-coupled receptor FFA1/GPR40. J. Cell. Physiol..

[184] Munkarah A (2016). Targeting of free fatty acid receptor 1 in EOC: a novel strategy to restrict the adipocyte-EOC dependence. Gynecol. Oncol..

[185] Zhang X, Yan G, Li Y, Zhu W, Wang H (2010). DC260126, a small-molecule antagonist of GPR40, improves insulin tolerance but not glucose tolerance in obese Zucker rats. Biomed. Pharmacother..

[186] Rubbino F (2022). GPR120 prevents colorectal adenocarcinoma progression by sustaining the mucosal barrier integrity. Sci. Rep..

[187] Wang X (2019). Fatty acid receptor GPR120 promotes breast cancer chemoresistance by upregulating ABC transporters expression and fatty acid synthesis. EBioMedicine.

[188] Watterson KR (2017). Probe-dependent negative allosteric modulators of the long-chain free fatty acid receptor FFA4. Mol. Pharm..

[189] Senatorov IS, Moniri NH (2018). The role of free-fatty acid receptor-4 (FFA4) in human cancers and cancer cell lines. Biochem. Pharmacol..

[190] Wu Q (2013). Identification of G-protein-coupled receptor 120 as a tumor-promoting receptor that induces angiogenesis and migration in human colorectal carcinoma. Oncogene.

[191] Liang P (2022). Effects of dietary omega-3 fatty acids on orthotopic prostate cancer progression, tumor associated macrophages, angiogenesis and T-cell activation-dependence on GPR120. Prostate Cancer Prostatic Dis..

[192] Hudson BD (2013). The pharmacology of TUG-891, a potent and selective agonist of the free fatty acid receptor 4 (FFA4/GPR120), demonstrates both potential opportunity and possible challenges to therapeutic agonism. Mol. Pharm..

[193] Lombardo M (2016). Discovery of benzofuran propanoic acid GPR120 agonists: from uHTS hit to mechanism-based pharmacodynamic effects. Bioorg. Med. Chem. Lett..

[194] Mao C (2023). Unsaturated bond recognition leads to biased signal in a fatty acid receptor. Science.

[195] Dong DD, Zhou H, Li G (2016). GPR78 promotes lung cancer cell migration and metastasis by activation of Galphaq-Rho GTPase pathway. BMB Rep..

[196] Misra UK, Pizzo SV (2010). Modulation of the unfolded protein response in prostate cancer cells by antibody-directed against the carboxyl-terminal domain of GRP78. Apoptosis.

[197] Lin XJ (2020). miR-936 suppresses cell proliferation, invasion, and drug resistance of laryngeal squamous cell carcinoma and targets GPR78. Front. Oncol..

[198] Park JH (2016). Fatty acid oxidation-driven Src links mitochondrial energy reprogramming and oncogenic properties in triple-negative breast cancer. Cell Rep..

[199] Jiang L (2015). Metabolic reprogramming during TGFbeta1-induced epithelial-to-mesenchymal transition. Oncogene.

[200] van der Weyden L (2017). Genome-wide in vivo screen identifies novel host regulators of metastatic colonization. Nature.

[201] Ly LK (2020). Bacterial steroid-17,20-desmolase is a taxonomically rare enzymatic pathway that converts prednisone to 1,4-androstanediene-3,11,17-trione, a metabolite that causes proliferation of prostate cancer cells. J. Steroid Biochem. Mol. Biol..

[202] Alauddin M (2020). Gut bacterial metabolite urolithin A decreases actin polymerization and migration in cancer cells. Mol. Nutr. Food Res..

[203] Bosco EE, Mulloy JC, Zheng Y (2009). Rac1 GTPase: a “Rac” of all trades. Cell Mol. Life Sci..

[204] Yamaguchi H, Condeelis J (2007). Regulation of the actin cytoskeleton in cancer cell migration and invasion. Biochim. Biophys. Acta.

[205] Bauer NN, Chen YW, Samant RS, Shevde LA, Fodstad O (2007). Rac1 activity regulates proliferation of aggressive metastatic melanoma. Exp. Cell Res..

[206] Kamai T (2004). Overexpression of RhoA, Rac1, and Cdc42 GTPases is associated with progression in testicular cancer. Clin. Cancer Res..

[207] Gimenez-Bastida JA, Avila-Galvez MA, Espin JC, Gonzalez-Sarrias A (2020). The gut microbiota metabolite urolithin A, but not other relevant urolithins, induces p53-dependent cellular senescence in human colon cancer cells. Food Chem. Toxicol..

[208] Shen H, Lu Z, Xu Z, Chen Z, Shen Z (2017). Associations among dietary non-fiber carbohydrate, ruminal microbiota and epithelium G-protein-coupled receptor, and histone deacetylase regulations in goats. Microbiome.

[209] Makki K, Deehan EC, Walter J, Backhed F (2018). The impact of dietary fiber on gut microbiota in host health and disease. Cell Host Microbe.

[210] Koh A, De Vadder F, Kovatcheva-Datchary P, Backhed F (2016). From dietary fiber to host physiology: short-chain fatty acids as key bacterial metabolites. Cell.

[211] Andrade-Oliveira V (2015). Gut bacteria products prevent AKI induced by ischemia-reperfusion. J. Am. Soc. Nephrol..

[212] Arpaia N (2013). Metabolites produced by commensal bacteria promote peripheral regulatory T-cell generation. Nature.

[213] Bultman, S. J. Interplay between diet, gut microbiota, epigenetic events, and colorectal cancer. *Mol. Nutr. Food Res*. 10.1002/mnfr.201500902 (2017). 10.1002/mnfr.201500902 PMC5161716 27138454

[214] Mahmood Z, Shukla Y (2010). Death receptors: targets for cancer therapy. Exp. Cell Res..

[215] Fujita PA (2011). The UCSC Genome Browser database: update 2011. Nucleic Acids Res..

[216] Liu X (2013). Regulation of microRNAs by epigenetics and their interplay involved in cancer. J. Exp. Clin. Cancer Res..

[217] Sampath D (2012). Histone deacetylases mediate the silencing of miR-15a, miR-16, and miR-29b in chronic lymphocytic leukemia. Blood.

[218] Atarashi K (2013). Treg induction by a rationally selected mixture of Clostridia strains from the human microbiota. Nature.

[219] Chang PV, Hao L, Offermanns S, Medzhitov R (2014). The microbial metabolite butyrate regulates intestinal macrophage function via histone deacetylase inhibition. Proc. Natl Acad. Sci. USA.

[220] Kim K (2019). Propionate of a microbiota metabolite induces cell apoptosis and cell cycle arrest in lung cancer. Mol. Med. Rep..

[221] Hogh RI (2020). Metabolism of short-chain fatty acid propionate induces surface expression of NKG2D ligands on cancer cells. FASEB J..

[222] Luu TH (2008). A phase II trial of vorinostat (suberoylanilide hydroxamic acid) in metastatic breast cancer: a California Cancer Consortium study. Clin. Cancer Res..

[223] Ramaswamy B (2012). Phase I-II study of vorinostat plus paclitaxel and bevacizumab in metastatic breast cancer: evidence for vorinostat-induced tubulin acetylation and Hsp90 inhibition in vivo. Breast Cancer Res. Treat..

[224] Stearns V (2013). Biomarker modulation following short-term vorinostat in women with newly diagnosed primary breast cancer. Clin. Cancer Res..

[225] Yardley DA (2013). Randomized phase II, double-blind, placebo-controlled study of exemestane with or without entinostat in postmenopausal women with locally recurrent or metastatic estrogen receptor-positive breast cancer progressing on treatment with a nonsteroidal aromatase inhibitor. J. Clin. Oncol..

[226] Munster P (2009). Clinical and biological effects of valproic acid as a histone deacetylase inhibitor on tumor and surrogate tissues: phase I/II trial of valproic acid and epirubicin/FEC. Clin. Cancer Res..

[227] Robertson FM (2013). The class I HDAC inhibitor Romidepsin targets inflammatory breast cancer tumor emboli and synergizes with paclitaxel to inhibit metastasis. J. Exp. Ther. Oncol..

[228] Duvic M (2007). Phase 2 trial of oral vorinostat (suberoylanilide hydroxamic acid, SAHA) for refractory cutaneous T-cell lymphoma (CTCL). Blood.

[229] Olsen EA (2007). Phase IIb multicenter trial of vorinostat in patients with persistent, progressive, or treatment refractory cutaneous T-cell lymphoma. J. Clin. Oncol..

[230] Mahalingam D (2014). Combined autophagy and HDAC inhibition: a phase I safety, tolerability, pharmacokinetic, and pharmacodynamic analysis of hydroxychloroquine in combination with the HDAC inhibitor vorinostat in patients with advanced solid tumors. Autophagy.

[231] Zibelman M (2015). Phase I study of the mTOR inhibitor ridaforolimus and the HDAC inhibitor vorinostat in advanced renal cell carcinoma and other solid tumors. Invest. N. Drugs.

[232] Kim KP (2015). First-in-human study of the toxicity, pharmacokinetics, and pharmacodynamics of CG200745, a pan-HDAC inhibitor, in patients with refractory solid malignancies. Invest. N. Drugs.

[233] Eigl BJ (2015). A phase II study of the HDAC inhibitor SB939 in patients with castration resistant prostate cancer: NCIC clinical trials group study IND195. Invest. N. Drugs.

[234] Damiano F (2020). Decanoic acid and not octanoic acid stimulates fatty acid synthesis in U87MG glioblastoma cells: a metabolomics study. Front. Neurosci..

[235] Weber DD (2020). Ketogenic diet in the treatment of cancer - where do we stand?. Mol. Metab..

[236] Poolchanuan P (2020). An anticonvulsive drug, valproic acid (valproate), has effects on the biosynthesis of fatty acids and polyketides in microorganisms. Sci. Rep..

[237] Møller N (2020). Ketone body, 3-hydroxybutyrate: minor metabolite - major medical manifestations. J. Clin. Endocrinol. Metab..

[238] Khodabakhshi A (2020). Feasibility, safety, and beneficial effects of MCT-based ketogenic diet for breast cancer treatment: a randomized controlled trial study. Nutr. Cancer.

[239] Zhang S, Xie C (2017). The role of OXCT1 in the pathogenesis of cancer as a rate-limiting enzyme of ketone body metabolism. Life Sci..

[240] Serhan CN (2014). Pro-resolving lipid mediators are leads for resolution physiology. Nature.

[241] Hsiao HM (2013). A novel anti-inflammatory and pro-resolving role for resolvin D1 in acute cigarette smoke-induced lung inflammation. PLoS ONE.

[242] Groeger AL (2010). Cyclooxygenase-2 generates anti-inflammatory mediators from omega-3 fatty acids. Nat. Chem. Biol..

[243] Lefils-Lacourtablaise J (2013). The eicosapentaenoic acid metabolite 15-deoxy-delta(12,14)-prostaglandin J3 increases adiponectin secretion by adipocytes partly via a PPARgamma-dependent mechanism. PLoS ONE.

[244] Musiek ES (2008). Electrophilic cyclopentenone neuroprostanes are anti-inflammatory mediators formed from the peroxidation of the omega-3 polyunsaturated fatty acid docosahexaenoic acid. J. Biol. Chem..

[245] Cipollina C (2014). Dual anti-oxidant and anti-inflammatory actions of the electrophilic cyclooxygenase-2-derived 17-oxo-DHA in lipopolysaccharide- and cigarette smoke-induced inflammation. Biochim. Biophys. Acta.

[246] de Jong E, Winkel P, Poelstra K, Prakash J (2011). Anticancer effects of 15d-prostaglandin-J2 in wild-type and doxorubicin-resistant ovarian cancer cells: novel actions on SIRT1 and HDAC. PLoS ONE.

[247] Doyle K, Fitzpatrick FA (2010). Redox signaling, alkylation (carbonylation) of conserved cysteines inactivates class I histone deacetylases 1, 2, and 3 and antagonizes their transcriptional repressor function. J. Biol. Chem..

[248] Codreanu SG, Zhang B, Sobecki SM, Billheimer DD, Liebler DC (2009). Global analysis of protein damage by the lipid electrophile 4-hydroxy-2-nonenal. Mol. Cell Proteom..

[249] Fritz KS (2011). 4-Hydroxynonenal inhibits SIRT3 via thiol-specific modification. Chem. Res. Toxicol..

[250] Ravindra KC, Narayan V, Lushington GH, Peterson BR, Prabhu KS (2012). Targeting of histone acetyltransferase p300 by cyclopentenone prostaglandin Delta(12)-PGJ(2) through covalent binding to Cys(1438). Chem. Res. Toxicol..

[251] Amarasekera M (2014). Epigenome-wide analysis of neonatal CD4(+) T-cell DNA methylation sites potentially affected by maternal fish oil supplementation. Epigenetics.

[252] Dierge E, Larondelle Y, Feron O (2020). Cancer diets for cancer patients: Lessons from mouse studies and new insights from the study of fatty acid metabolism in tumors. Biochimie.

[253] Dimri M, Bommi PV, Sahasrabuddhe AA, Khandekar JD, Dimri GP (2010). Dietary omega-3 polyunsaturated fatty acids suppress expression of EZH2 in breast cancer cells. Carcinogenesis.

[254] Mandal CC, Ghosh-Choudhury T, Yoneda T, Choudhury GG, Ghosh-Choudhury N (2010). Fish oil prevents breast cancer cell metastasis to bone. Biochem. Biophys. Res. Commun..

[255] Xu H, Niu M, Yuan X, Wu K, Liu A (2020). CD44 as a tumor biomarker and therapeutic target. Exp. Hematol. Oncol..

[256] Hwang JK (2017). DHA blocks TPA-induced cell invasion by inhibiting MMP-9 expression via suppression of the PPAR-gamma/NF-kappaB pathway in MCF-7 cells. Oncol. Lett..

[257] Song NY, Na HK, Baek JH, Surh YJ (2014). Docosahexaenoic acid inhibits insulin-induced activation of sterol regulatory-element binding protein 1 and cyclooxygenase-2 expression through upregulation of SIRT1 in human colon epithelial cells. Biochem. Pharmacol..

[258] Jung SB (2013). Docosahexaenoic acid improves vascular function via up-regulation of SIRT1 expression in endothelial cells. Biochem. Biophys. Res. Commun..

[259] Cho Y (2011). A chemoprotective fish oil- and pectin-containing diet temporally alters gene expression profiles in exfoliated rat colonocytes throughout oncogenesis. J. Nutr..

[260] Kolar SS (2007). Synergy between docosahexaenoic acid and butyrate elicits p53-independent apoptosis via mitochondrial Ca(2+) accumulation in colonocytes. Am. J. Physiol. Gastrointest. Liver Physiol..

[261] Turk HF (2011). Linoleic acid and butyrate synergize to increase Bcl-2 levels in colonocytes. Int. J. Cancer.

[262] Zhang SL (2021). Pectin supplement significantly enhanced the anti-PD-1 efficacy in tumor-bearing mice humanized with gut microbiota from patients with colorectal cancer. Theranostics.

[263] Cho Y (2014). Colon cancer cell apoptosis is induced by combined exposure to the n-3 fatty acid docosahexaenoic acid and butyrate through promoter methylation. Exp. Biol. Med..

[264] Shah MS (2011). Integrated microRNA and mRNA expression profiling in a rat colon carcinogenesis model: effect of a chemo-protective diet. Physiol. Genomics.

[265] Yao L (2015). Omega-3 polyunsaturated fatty acids upregulate 15-PGDH expression in cholangiocarcinoma cells by inhibiting miR-26a/b expression. Cancer Res..

[266] Myung SJ (2006). 15-Hydroxyprostaglandin dehydrogenase is an in vivo suppressor of colon tumorigenesis. Proc. Natl Acad. Sci. USA.

[267] Huang G (2008). 15-Hydroxyprostaglandin dehydrogenase is a target of hepatocyte nuclear factor 3beta and a tumor suppressor in lung cancer. Cancer Res..

[268] Na HK (2011). 15-Hydroxyprostaglandin dehydrogenase as a novel molecular target for cancer chemoprevention and therapy. Biochem. Pharmacol..

[269] Hannafon BN (2015). Exosome-mediated microRNA signaling from breast cancer cells is altered by the anti-angiogenesis agent docosahexaenoic acid (DHA). Mol. Cancer.

[270] Nair SV, Ziaullah, Rupasinghe HP (2014). Fatty acid esters of phloridzin induce apoptosis of human liver cancer cells through altered gene expression. PLoS ONE.

[271] Swygert SG, Peterson CL (2014). Chromatin dynamics: interplay between remodeling enzymes and histone modifications. Biochim. Biophys. Acta.

[272] Yazbeck VY, Grant S (2015). Romidepsin for the treatment of non-Hodgkin’s lymphoma. Expert Opin. Invest. Drugs.

[273] Richon VM, Garcia-Vargas J, Hardwick JS (2009). Development of vorinostat: current applications and future perspectives for cancer therapy. Cancer Lett..

[274] Bavaresco L, Lucini L, Busconi M, Flamini R, De Rosso M (2016). Wine resveratrol: from the ground up. Nutrients.

[275] Nowicki A (2020). The effect of 3’-hydroxy-3,4,5,4’-tetramethoxy-stilbene, the metabolite of the resveratrol analogue DMU-212, on the motility and proliferation of ovarian cancer cells. Int. J. Mol. Sci..

[276] Ji B (2018). GPR56 promotes proliferation of colorectal cancer cells and enhances metastasis via epithelialmesenchymal transition through PI3K/AKT signaling activation. Oncol. Rep..

[277] Jin HR, Du CH, Wang CZ, Yuan CS, Du W (2019). Ginseng metabolite protopanaxadiol interferes with lipid metabolism and induces endoplasmic reticulum stress and p53 activation to promote cancer cell death. Phytother. Res..

[278] Rathmell JC, Newgard CB (2009). Biochemistry. A glucose-to-gene link. Science.

[279] Wellen KE, Thompson CB (2012). A two-way street: reciprocal regulation of metabolism and signalling. Nat. Rev. Mol. Cell Biol..

[280] Alli PM, Pinn ML, Jaffee EM, McFadden JM, Kuhajda FP (2005). Fatty acid synthase inhibitors are chemopreventive for mammary cancer in neu-N transgenic mice. Oncogene.

[281] Li Z (2021). Acetyl-CoA synthetase 2: a critical linkage in obesity-induced tumorigenesis in myeloma. Cell Metab..

[282] Kargbo RB (2019). Inhibition of ACSS2 for treatment of cancer and neuropsychiatric diseases. ACS Med. Chem. Lett..

[283] Galdieri L, Vancura A (2012). Acetyl-CoA carboxylase regulates global histone acetylation. J. Biol. Chem..

[284] Gouw AM (2019). The MYC oncogene cooperates with sterol-regulated element-binding protein to regulate lipogenesis essential for neoplastic growth. Cell Metab..

[285] Svensson RU (2016). Inhibition of acetyl-CoA carboxylase suppresses fatty acid synthesis and tumor growth of non-small-cell lung cancer in preclinical models. Nat. Med..

[286] Harriman G (2016). Acetyl-CoA carboxylase inhibition by ND-630 reduces hepatic steatosis, improves insulin sensitivity, and modulates dyslipidemia in rats. Proc. Natl Acad. Sci. USA.

[287] McDonnell E (2016). Lipids reprogram metabolism to become a major carbon source for histone acetylation. Cell Rep..

[288] Qiu J (2019). Acetate promotes T cell effector function during glucose restriction. Cell Rep..

[289] Lu M (2019). ACOT12-dependent alteration of acetyl-CoA drives hepatocellular carcinoma metastasis by epigenetic induction of epithelial-mesenchymal transition. Cell Metab..

[290] Prabakaran S, Lippens G, Steen H, Gunawardena J (2012). Post-translational modification: nature’s escape from genetic imprisonment and the basis for dynamic information encoding. Wiley Interdiscip. Rev. Syst. Biol. Med..

[291] Wysocka J (2005). WDR5 associates with histone H3 methylated at K4 and is essential for H3 K4 methylation and vertebrate development. Cell.

[292] Wysocka J (2006). A PHD finger of NURF couples histone H3 lysine 4 trimethylation with chromatin remodelling. Nature.

[293] Berdasco M, Esteller M (2010). Aberrant epigenetic landscape in cancer: how cellular identity goes awry. Dev. Cell.

[294] Fullgrabe J, Kavanagh E, Joseph B (2011). Histone onco-modifications. Oncogene.

[295] Choudhary C, Weinert BT, Nishida Y, Verdin E, Mann M (2014). The growing landscape of lysine acetylation links metabolism and cell signalling. Nat. Rev. Mol. Cell Biol..

[296] Martin C, Zhang Y (2007). Mechanisms of epigenetic inheritance. Curr. Opin. Cell Biol..

[297] Ruthenburg AJ, Li H, Patel DJ, Allis CD (2007). Multivalent engagement of chromatin modifications by linked binding modules. Nat. Rev. Mol. Cell Biol..

[298] Tan M (2011). Identification of 67 histone marks and histone lysine crotonylation as a new type of histone modification. Cell.

[299] Liu Z (2015). Integrative chemical biology approaches for identification and characterization of “erasers” for fatty-acid-acylated lysine residues within proteins. Angew. Chem. Int. Ed. Engl..

[300] Farazi TA, Waksman G, Gordon JI (2001). The biology and enzymology of protein N-myristoylation. J. Biol. Chem..

[301] Shanmugam MK (2018). Role of novel histone modifications in cancer. Oncotarget.

[302] Elsheikh SE (2009). Global histone modifications in breast cancer correlate with tumor phenotypes, prognostic factors, and patient outcome. Cancer Res..

[303] Moffett JR, Puthillathu N, Vengilote R, Jaworski DM, Namboodiri AM (2020). Acetate revisited: a key biomolecule at the nexus of metabolism, epigenetics, and oncogenesis - Part 2: Acetate and ACSS2 in health and disease. Front. Physiol..

[304] Chang SC (2020). A gut butyrate-producing bacterium *Butyricicoccus pullicaecorum* regulates short-chain fatty acid transporter and receptor to reduce the progression of 1,2-dimethylhydrazine-associated colorectal cancer. Oncol. Lett..

[305] Wang S (2015). Metabolism. Lysosomal amino acid transporter SLC38A9 signals arginine sufficiency to mTORC1. Science.

[306] Gu X (2017). SAMTOR is an S-adenosylmethionine sensor for the mTORC1 pathway. Science.

[307] Findlay GM, Yan L, Procter J, Mieulet V, Lamb RF (2007). A MAP4 kinase related to Ste20 is a nutrient-sensitive regulator of mTOR signalling. Biochem. J..

[308] Han JM (2012). Leucyl-tRNA synthetase is an intracellular leucine sensor for the mTORC1-signaling pathway. Cell.

[309] Zoncu R (2011). mTORC1 senses lysosomal amino acids through an inside-out mechanism that requires the vacuolar H(+)-ATPase. Science.

[310] Sancak Y (2010). Ragulator-Rag complex targets mTORC1 to the lysosomal surface and is necessary for its activation by amino acids. Cell.

[311] Sancak Y (2008). The Rag GTPases bind raptor and mediate amino acid signaling to mTORC1. Science.

[312] Guan KL (2008). Regulation of TORC1 by Rag GTPases in nutrient response. Nat. Cell Biol..

[313] Ljungdahl PO, Daignan-Fornier B (2012). Regulation of amino acid, nucleotide, and phosphate metabolism in Saccharomyces cerevisiae. Genetics.

[314] Bar-Peled L (2013). A Tumor suppressor complex with GAP activity for the Rag GTPases that signal amino acid sufficiency to mTORC1. Science.

[315] Wang X, Proud CG (2006). The mTOR pathway in the control of protein synthesis. Physiology.

[316] Meng D (2020). Glutamine and asparagine activate mTORC1 independently of Rag GTPases. J. Biol. Chem..

[317] Meunier G (2020). Antileukemic activity of the VPS34-IN1 inhibitor in acute myeloid leukemia. Oncogenesis.

[318] Giannikou K (2021). Subependymal giant cell astrocytomas are characterized by mTORC1 hyperactivation, a very low somatic mutation rate, and a unique gene expression profile. Mod. Pathol..

[319] Perez, R. E. et al. Prolyl endopeptidase inhibitor Y-29794 blocks the IRS1-AKT-mTORC1 pathway and inhibits survival and in vivo tumor growth of triple-negative breast cancer. *Cancer Biol. Ther.* **21**, 1033–1040 (2020). 10.1080/15384047.2020.1824989 PMC7678932 33044914

[320] Soliman GA (2020). The synergistic effect of an ATP-competitive inhibitor of mTOR and metformin on pancreatic tumor. Growth Curr. Dev. Nutr..

[321] Nakano, T. et al. mTOR inhibition ablates cisplatin-resistant salivary gland cancer stem cells. *J. Dent. Res.* **100**, 377–386 (2020). 10.1177/0022034520965141 PMC7989140 33073679

[322] Guan Y (2020). Combined treatment of mitoxantrone sensitizes breast cancer cells to rapalogs through blocking eEF-2K-mediated activation of Akt and autophagy. Cell Death Dis..

[323] Digby JE (2010). Anti-inflammatory effects of nicotinic acid in adipocytes demonstrated by suppression of fractalkine, RANTES, and MCP-1 and upregulation of adiponectin. Atherosclerosis.

[324] Gambhir D (2012). GPR109A as an anti-inflammatory receptor in retinal pigment epithelial cells and its relevance to diabetic retinopathy. Invest. Ophthalmol. Vis. Sci..

[325] Geng HW, Yin FY, Zhang ZF, Gong X, Yang Y (2021). Butyrate suppresses glucose metabolism of colorectal cancer cells via GPR109a-AKT signaling pathway and enhances chemotherapy. Front. Mol. Biosci..

[326] Ferrere G (2021). Ketogenic diet and ketone bodies enhance the anticancer effects of PD-1 blockade. JCI Insight.

[327] Venkateswaran N (2019). MYC promotes tryptophan uptake and metabolism by the kynurenine pathway in colon cancer. Genes Dev..

[328] Irukayama-Tomobe Y (2009). Aromatic D-amino acids act as chemoattractant factors for human leukocytes through a G protein-coupled receptor, GPR109B. Proc. Natl Acad. Sci. USA.

[329] Sleiman, P. Common variants at five new loci associated with early-onset inflammatory bowel disease. *Nat. Genet*. **41**, 1335–1340 (2009). 10.1038/ng.489 PMC3267927 19915574

[330] Men LJ (2018). Down regulation of G protein-coupled receptor 137 expression inhibits proliferation and promotes apoptosis in leukemia cells. Cancer Cell Int..

[331] Xu L, Begum S, Hearn JD, Hynes RO (2006). GPR56, an atypical G protein-coupled receptor, binds tissue transglutaminase, TG2, and inhibits melanoma tumor growth and metastasis. Proc. Natl Acad. Sci. USA.

[332] Luo R (2011). G protein-coupled receptor 56 and collagen III, a receptor-ligand pair, regulates cortical development and lamination. Proc. Natl Acad. Sci. USA.

[333] Kausar T (2011). Clinical significance of GPR56, transglutaminase 2, and NF-kappaB in esophageal squamous cell carcinoma. Cancer Invest..

[334] Shashidhar S (2005). GPR56 is a GPCR that is overexpressed in gliomas and functions in tumor cell adhesion. Oncogene.

[335] Chiang NY (2017). GPR56/ADGRG1 activation promotes melanoma cell migration via NTF dissociation and CTF-mediated Galpha12/13/RhoA signaling. J. Invest. Dermatol..

[336] Saito Y (2013). Maintenance of the hematopoietic stem cell pool in bone marrow niches by EVI1-regulated GPR56. Leukemia.

[337] Yang L (2011). GPR56 regulates VEGF production and angiogenesis during melanoma progression. Cancer Res..

[338] Xu L (2010). GPR56 plays varying roles in endogenous cancer progression. Clin. Exp. Metastasis.

[339] Zhang S (2019). GPR56 drives colorectal tumor growth and promotes drug resistance through upregulation of MDR1 expression via a RhoA-mediated mechanism. Mol. Cancer Res..

[340] Iguchi T (2008). Orphan G protein-coupled receptor GPR56 regulates neural progenitor cell migration via a G alpha 12/13 and Rho pathway. J. Biol. Chem..

[341] Stoveken HM, Larsen SD, Smrcka AV, Tall GG (2018). Gedunin- and khivorin-derivatives are small-molecule partial agonists for adhesion G protein-coupled receptors GPR56/ADGRG1 and GPR114/ADGRG5. Mol. Pharm..

[342] Ohta S (2015). Agonistic antibodies reveal the function of GPR56 in human glioma U87-MG cells. Biol. Pharm. Bull..

[343] Ji X (2018). xCT (SLC7A11)-mediated metabolic reprogramming promotes non-small cell lung cancer progression. Oncogene.

[344] Yu LJ, Wall BA, Wangari-Talbot J, Chen S (2017). Metabotropic glutamate receptors in cancer. Neuropharmacology.

[345] Kappler M (2019). Causes and consequences of a glutamine induced normoxic HIF1 activity for the tumor metabolism. Int. J. Mol. Sci..

[346] Cobler L, Zhang H, Suri P, Park C, Timmerman LA (2018). xCT inhibition sensitizes tumors to gamma-radiation via glutathione reduction. Oncotarget.

[347] Magri J (2021). Tumor-associated antigen xCT and mutant-p53 as molecular targets for new combinatorial antitumor strategies. Cells.

[348] Tarragó-Celada J (2021). Cysteine and folate metabolism are targetable vulnerabilities of metastatic colorectal cancer. Cancers.

[349] Koppula P, Zhuang L, Gan B (2021). Cystine transporter SLC7A11/xCT in cancer: ferroptosis, nutrient dependency, and cancer therapy. Protein Cell.

[350] Lukey MJ, Katt WP, Cerione RA (2017). Targeting amino acid metabolism for cancer therapy. Drug Discov. Today.

[351] Hirshfield KM (2013). Metabotropic glutamate receptor 1 expression and its polymorphic variants associate with breast cancer phenotypes. PLoS ONE.

[352] Herner A (2011). Glutamate increases pancreatic cancer cell invasion and migration via AMPA receptor activation and Kras-MAPK signaling. Int. J. Cancer.

[353] Bhutia YD, Ganapathy V (2016). Glutamine transporters in mammalian cells and their functions in physiology and cancer. Biochim. Biophys. Acta.

[354] Broer A (2019). Ablation of the ASCT2 (SLC1A5) gene encoding a neutral amino acid transporter reveals transporter plasticity and redundancy in cancer cells. J. Biol. Chem..

[355] Schulte ML (2018). Pharmacological blockade of ASCT2-dependent glutamine transport leads to antitumor efficacy in preclinical models. Nat. Med..

[356] Lee B (2020). Integrated RNA and metabolite profiling of urine liquid biopsies for prostate cancer biomarker discovery. Sci. Rep..

[357] Ohkawa M (2011). Oncogenicity of L-type amino-acid transporter 1 (LAT1) revealed by targeted gene disruption in chicken DT40 cells: LAT1 is a promising molecular target for human cancer therapy. Biochem. Biophys. Res. Commun..

[358] Fan X (2010). Impact of system L amino acid transporter 1 (LAT1) on proliferation of human ovarian cancer cells: a possible target for combination therapy with anti-proliferative aminopeptidase inhibitors. Biochem. Pharmacol..

[359] Choudhari SK, Chaudhary M, Bagde S, Gadbail AR, Joshi V (2013). Nitric oxide and cancer: a review. World J. Surg. Oncol..

[360] Ridnour LA (2008). Molecular mechanisms for discrete nitric oxide levels in cancer. Nitric Oxide.

[361] Piazza M, Guillemette JG, Dieckmann T (2015). Dynamics of nitric oxide synthase–calmodulin interactions at physiological calcium concentrations. Biochemistry.

[362] Stryzewski W, Flejsierowiczowa Z, Kabschowa B (1966). [Situation of epileptic persons in some industrial plants in Poznan]. Med. Pract..

[363] Metzen E, Zhou J, Jelkmann W, Fandrey J, Brune B (2003). Nitric oxide impairs normoxic degradation of HIF-1alpha by inhibition of prolyl hydroxylases. Mol. Biol. Cell.

[364] Walczak K (2020). A tryptophan metabolite, 8-hydroxyquinaldic acid, exerts antiproliferative and anti-migratory effects on colorectal cancer cells. Molecules.

[365] Kovacs T (2019). Cadaverine, a metabolite of the microbiome, reduces breast cancer aggressiveness through trace amino acid receptors. Sci. Rep..

[366] Wu J, Wu M, Wu Q (2020). Identification of potential metabolite markers for colon cancer and rectal cancer using serum metabolomics. J. Clin. Lab. Anal..

[367] Ni J, Xu L, Li W, Zheng C, Wu L (2019). Targeted metabolomics for serum amino acids and acylcarnitines in patients with lung cancer. Exp. Ther. Med..

[368] Geiger R (2016). L-Arginine Modulates T cell metabolism and enhances survival and anti-tumor activity. Cell.

[369] Jaendling A, McFarlane RJ (2010). Biological roles of translin and translin-associated factor-X: RNA metabolism comes to the fore. Biochem. J..

[370] Atta IS (2021). Efficacy of expressions of Arg-1, Hep Par-1, and CK19 in the diagnosis of the primary hepatocellular carcinoma subtypes and exclusion of the metastases. Histol. Histopathol..

[371] Brown M (2012). The differential effects of statins on the metastatic behaviour of prostate cancer. Br. J. Cancer.

[372] Kakehashi A (2020). Accumulation of 8-hydroxydeoxyguanosine, L-arginine and Glucose Metabolites By Liver Tumor Cells Are The Important Characteristic Features Of Metabolic Syndrome And Non-alcoholic Steatohepatitis-associated Hepatocarcinogenesis. Int. J. Mol. Sci..

[373] Zhu M, Wang Y, Wang F, Li L, Qiu X (2021). CircFBXL5 promotes the 5-FU resistance of breast cancer via modulating miR-216b/HMGA2 axis. Cancer Cell Int..

[374] Gupta VA (2021). Venetoclax sensitivity in multiple myeloma is associated with B-cell gene expression. Blood.

[375] Badgley MA (2020). Cysteine depletion induces pancreatic tumor ferroptosis in mice. Science.

[376] Pietrocola F, Galluzzi L, Bravo-San Pedro JM, Madeo F, Kroemer G (2015). Acetyl coenzyme A: a central metabolite and second messenger. Cell Metab..

[377] Son SM (2019). Leucine signals to mTORC1 via its metabolite acetyl-coenzyme A. Cell Metab..

[378] Panosyan EH, Lin HJ, Koster J, Lasky JL (2017). In search of druggable targets for GBM amino acid metabolism. BMC Cancer.

[379] Tonjes M (2013). BCAT1 promotes cell proliferation through amino acid catabolism in gliomas carrying wild-type IDH1. Nat. Med..

[380] Zheng YH (2016). BCAT1, a key prognostic predictor of hepatocellular carcinoma, promotes cell proliferation and induces chemoresistance to cisplatin. Liver Int..

[381] Hutson SM (2016). Leucine metabolism in T cell activation: mTOR signaling and beyond. Adv. Nutr..

[382] Ananieva EA, Patel CH, Drake CH, Powell JD, Hutson SM (2014). Cytosolic branched chain aminotransferase (BCATc) regulates mTORC1 signaling and glycolytic metabolism in CD4+ T cells. J. Biol. Chem..

[383] Mayers JR (2016). Tissue of origin dictates branched-chain amino acid metabolism in mutant Kras-driven cancers. Science.

[384] Zhang B (2022). Targeting BCAT1 combined with alpha-ketoglutarate triggers metabolic synthetic lethality in glioblastoma. Cancer Res..

[385] Hattori A (2017). Cancer progression by reprogrammed BCAA metabolism in myeloid leukaemia. Nature.

[386] Wu Z (2015). TPO-induced metabolic reprogramming drives liver metastasis of colorectal cancer CD110+ tumor-initiating cells. Cell Stem Cell.

[387] Drew HR, Dickerson RE (1981). Structure of a B-DNA dodecamer. III. Geometry of hydration. J. Mol. Biol..

[388] Nakanishi S, Cleveland JL (2016). Targeting the polyamine-hypusine circuit for the prevention and treatment of cancer. Amino Acids.

[389] Liu R (2017). Plasma N-acetylputrescine, cadaverine and 1,3-diaminopropane: potential biomarkers of lung cancer used to evaluate the efficacy of anticancer drugs. Oncotarget.

[390] Nowotarski SL, Woster PM, Casero RA (2013). Polyamines and cancer: implications for chemotherapy and chemoprevention. Expert Rev. Mol. Med..

[391] Xu H (2016). Polyamine metabolites profiling for characterization of lung and liver cancer using an LC-tandem MS method with multiple statistical data mining strategies: discovering potential cancer biomarkers in human plasma and urine. Molecules.

[392] Dominguez D, Ye C, Geng Z, Chen S, Zhang B (2016). Exogenous IL-33 restores dendritic cell activation and maturation in established cancer. J. Immunol..

[393] Lee YQ, Rajadurai P, Abas F, Othman I, Naidu R (2021). Proteomic analysis on anti-proliferative and apoptosis effects of curcumin analog, 1,5-bis(4-hydroxy-3-methyoxyphenyl)-1,4-pentadiene-3-one-treated human glioblastoma and neuroblastoma cells. Front. Mol. Biosci..

[394] Zheng ZQ (2020). Long noncoding RNA TINCR-mediated regulation of Acetyl-CoA metabolism promotes nasopharyngeal carcinoma progression and chemoresistance. Cancer Res..

[395] Wang WJ (2021). Overview of serpin B9 and its roles in cancer (Review). Oncol. Rep..

[396] Mahmood K, Emadi A (2021). 1-C metabolism-serine, glycine, folates-in acute myeloid leukemia. Pharmacology.

[397] Hakobyan S, Loeffler-Wirth H, Arakelyan A, Binder H, Kunz M (2021). A transcriptome-wide isoform landscape of melanocytic nevi and primary melanomas identifies gene isoforms associated with malignancy. Int. J. Mol. Sci..

[398] Chaneton B, Gottlieb E (2012). Rocking cell metabolism: revised functions of the key glycolytic regulator PKM2 in cancer. Trends Biochem. Sci..

[399] Yang W, Lu Z (2015). Pyruvate kinase M2 at a glance. J. Cell Sci..

[400] Cha PH (2021). APC loss induces Warburg effect via increased PKM2 transcription in colorectal cancer. Br. J. Cancer.

[401] Wang X (2020). Exosome-delivered circRNA promotes glycolysis to induce chemoresistance through the miR-122-PKM2 axis in colorectal cancer. Mol. Oncol..

[402] Maria RM, Altei WF, Selistre-de-Araujo HS, Colnago LA (2017). Effects of doxorubicin, cisplatin, and tamoxifen on the metabolic profile of human breast cancer MCF-7 cells as determined by (1)H high-resolution magic angle spinning nuclear magnetic resonance. Biochemistry.

[403] Hamabe A, Konno M, Tanuma N, Shima H, Ishii H (2014). Role of pyruvate kinase M2 in transcriptional regulation leading to epithelial-mesenchymal transition. Proc. Natl Acad. Sci. USA.

[404] Ensor CM, Holtsberg FW, Bomalaski JS, Clark MA (2002). Pegylated arginine deiminase (ADI-SS PEG20,000 mw) inhibits human melanomas and hepatocellular carcinomas in vitro and in vivo. Cancer Res..

[405] Miraki-Moud F (2015). Arginine deprivation using pegylated arginine deiminase has activity against primary acute myeloid leukemia cells in vivo. Blood.

[406] Szlosarek PW (2017). Arginine deprivation with pegylated arginine deiminase in patients with argininosuccinate synthetase 1-deficient malignant pleural mesothelioma: a randomized clinical trial. JAMA Oncol..

[407] He Y (2019). Metabolic intermediates in tumorigenesis and progression. Int. J. Biol. Sci..

[408] Ulrey CL, Liu L, Andrews LG, Tollefsbol TO (2005). The impact of metabolism on DNA methylation. Hum. Mol. Genet..

[409] Stipanuk MH (2020). Metabolism of sulfur-containing amino acids: how the body copes with excess methionine, cysteine, and sulfide. J. Nutr..

[410] Williams KT, Schalinske KL (2007). New insights into the regulation of methyl group and homocysteine metabolism. J. Nutr..

[411] Borrego SL (2016). Metabolic changes associated with methionine stress sensitivity in MDA-MB-468 breast cancer cells. Cancer Metab..

[412] Maddocks OD, Labuschagne CF, Adams PD, Vousden KH (2016). Serine metabolism supports the methionine cycle and DNA/RNA methylation through de novo ATP synthesis in cancer cells. Mol. Cell.

[413] Oosterveer MH (2012). LRH-1-dependent glucose sensing determines intermediary metabolism in liver. J. Clin. Invest..

[414] Wang Z (2019). Methionine is a metabolic dependency of tumor-initiating cells. Nat. Med..

[415] Tabe Y, Lorenzi PL, Konopleva M (2019). Amino acid metabolism in hematologic malignancies and the era of targeted therapy. Blood.

[416] Couturier MA (2015). Cerebral venous thrombosis in adult patients with acute lymphoblastic leukemia or lymphoblastic lymphoma during induction chemotherapy with l-asparaginase: the GRAALL experience. Am. J. Hematol..

[417] Jaccard A (2011). Efficacy of L-asparaginase with methotrexate and dexamethasone (AspaMetDex regimen) in patients with refractory or relapsing extranodal NK/T-cell lymphoma, a phase 2 study. Blood.

